# Widely accessible method for 3D microflow mapping at high spatial and temporal resolutions

**DOI:** 10.1038/s41378-022-00404-z

**Published:** 2022-07-01

**Authors:** Evan Lammertse, Nikhil Koditala, Martin Sauzade, Hongxiao Li, Qiang Li, Luc Anis, Jun Kong, Eric Brouzes

**Affiliations:** 1 Department of Biomedical Engineering, Stony Brook University, Stony Brook, NY 11794 USA; 2 Department of Mathematics and Statistics, Department of Computer Science, Georgia State University, Atlanta, GA 30302 USA; 3 Laufer Center for Physical and Quantitative Biology, Stony Brook University, Stony Brook, NY 11794 USA; 4 Cancer Center, Stony Brook School of Medicine, Stony Brook, NY 11794 USA; 5 Institute for Engineering Driven Medicine, Stony Brook University, Stony Brook, NY 11794 USA

**Keywords:** Applied optics, Engineering

## Abstract

Advances in microfluidic technologies rely on engineered 3D flow patterns to manipulate samples at the microscale. However, current methods for mapping flows only provide limited 3D and temporal resolutions or require highly specialized optical set-ups. Here, we present a simple defocusing approach based on brightfield microscopy and open-source software to map micro-flows in 3D at high spatial and temporal resolution. Our workflow is both integrated in ImageJ and modular. We track seed particles in 2D before classifying their Z-position using a reference library. We compare the performance of a traditional cross-correlation method and a deep learning model in performing the classification step. We validate our method on three highly relevant microfluidic examples: a channel step expansion and displacement structures as single-phase flow examples, and droplet microfluidics as a two-phase flow example. First, we elucidate how displacement structures efficiently shift large particles across streamlines. Second, we reveal novel recirculation structures and folding patterns in the internal flow of microfluidic droplets. Our simple and widely accessible brightfield technique generates high-resolution flow maps and it will address the increasing demand for controlling fluids at the microscale by supporting the efficient design of novel microfluidic structures.

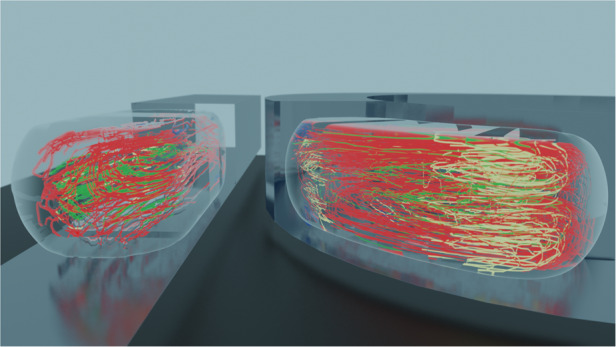

## Introduction

Microfluidic advances increasingly rely on engineered 3D flow patterns to manipulate samples at the micronscale. Numerical methods can provide insights into flow patterns; however, complex driving forces, conduit geometries, and fluid/surface interactions can preclude the accurate numerical simulation of microflows. It is also critical to validate flow topologies experimentally, which requires high-resolution 3D flow mapping techniques. Micro-particle image velocimetry (µPIV) is the “gold standard” for mapping single^1–3^ and multi-phase^4–6^ microflows. It cross-correlates local patterns created by densely seeded particles, and it is thus inherently a 2-dimensional method with a Z-resolution limited to several µm^4,7^. The flow between data slices can be calculated *via* the continuity equation^8,9^ at a high computing cost. Methods to improve PIV resolution include multiple camera set-ups^10–13^ or controlled sample illumination with a confocal disk^7,9^. In sum, added cost and complexity make high-resolution PIV techniques inadequate for broader adoption by the scientific community.

In contrast, single-particle tracking provides sub-pixel resolution in 2D that can be expanded into the third dimension by comparing particle images to a reference library of defocused patterns at known depths^14–16^. Defocusing micro-particle tracking velocimetry (µPTV) uses this approach to track tracer particles in microflows^14,17,18^. µPTV has been mostly implemented with fluorescence microscopy combined with either a 3-pinhole aperture^12^ or a cylindrical lens^19^ that create an asymmetric defocusing pattern. In these approaches, the low fluorescence signal is further attenuated by the additional optical components and the use of low numerical aperture (NA) objectives that provide a large depth of field at the expense of light collection. Overall, fluorescence microscopy and customized set-ups have decreased the utility of defocusing µPTV because they provide limited temporal resolution and require dedicated or elaborate^17^ optical set-ups.

Here, we establish a high-resolution µPTV approach based on a simple brightfield microscope set-up that alleviates current limitations. Our method is further made accessible by using open-source software (Fiji^20^) and plugin (TrackMate^21^) to track seed particles in 2D. In our modular workflow, the classification step can be performed by different methods. Deep learning models have proven very useful in classification problems in many different situations, and we develop and compare a deep learning model to the more classic cross-correlation method^14,18^. We measure the performance of the two classification approaches on experimental data. We then validate and demonstrate the usefulness of our 3D flow mapping method by characterizing the flow pattern of three representative microfluidic examples. First, we map the velocity field at a channel step expansion and then evaluate continuity error. Second, we demonstrate how our mapping method elucidates the working principles of displacement structures that allow for perfect particle trapping^22^. Third, we generate high-resolution maps of the internal flow of microfluidic droplets in a straight channel and in a curved channel known to induce mixing. Those real-life examples emphasize the utility of analyzing pathlines in addition to velocity fields to provide a detailed flow map. All experimental data can be explored in 3D with an online viewer at https://www.stonybrook.edu/commcms/defocusing_3D_mapping/index.html.

## Results

### Strategy

**Fig. 1:**
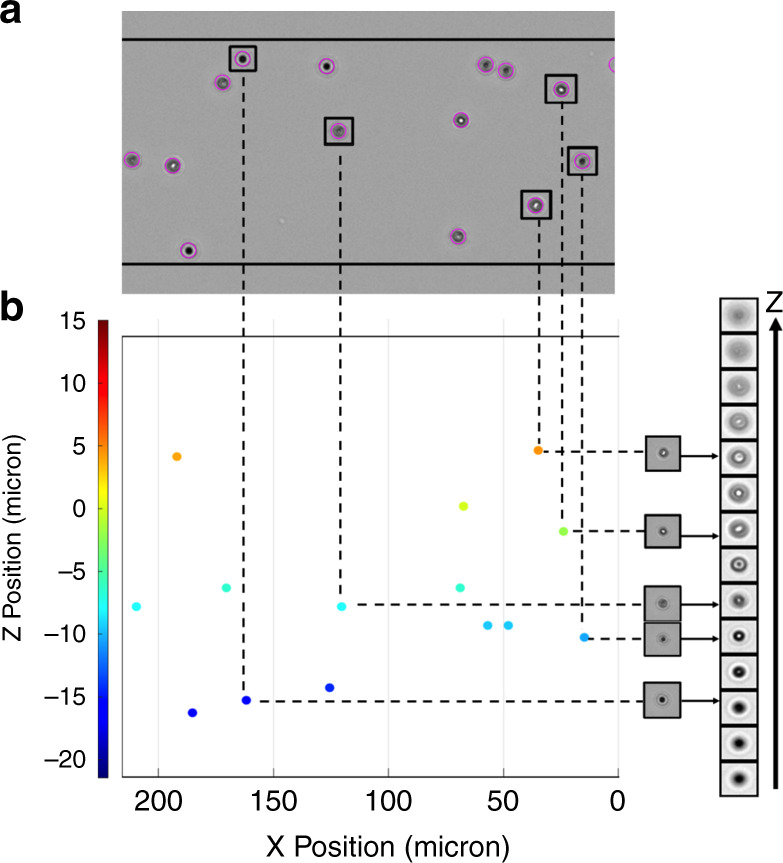
Brightfield defocusing strategy. **a** TrackMate, an ImageJ plugin, detects beads as “spots” (purple circles, *n* = 14) from high-speed video frames. It links spots from consecutive frames into 2D tracks (not shown here). **b** Particle coordinates are extended to 3D by image classification against a reference library of defocus patterns at known Z-location using either a cross-correlation algorithm or a deep learning model. Both approaches provide continuous positions based on discrete classes in the reference image set

Our novel technique to map microflows in 3D and at high resolution uses widely accessible brightfield microscopy and open-source algorithms. Efficient determination of Z-position relies on an optimal asymmetric defocusing pattern with the largest cone angle and deepest field of view (SI Appendix, Fig. S1). We use 3 μm diameter polystyrene microbeads to provide ideal defocusing patterns and sufficient pixels for efficient Z-prediction without off-center effects. We adjust the correction ring of the 20x/0.45NA objective to 0.2 mm to introduce spherical aberrations and remove ambiguity about the focal plane (SI Appendix, Fig. S2). Our two-step strategy first uses TrackMate^21^, a GUI-based particle tracking tool included in the Fiji distribution of ImageJ, to provide lateral (XY) particle trajectories. Particle density influences the number of “collisions” between seed particles. Higher seed densities yield more tracking data for a given video length and thus a higher resolution of the flow field; however, overlapping or near-overlapping particle events become more common. This challenges both TrackMate’s spot detection and particle linking algorithms. These errors require manual corrections to fix track defects if long pathlines are desired or to delete erroneous spots if velocity vectors are desired. Particle linking errors are more labor intensive to correct. We experimentally determined that a particle image density lower than 0.01 (pixel/pixel) provides a good compromise between data density and manual input (*SI Appendix* Seed Density Analysis, Table S1 and Fig. S3). Second, we expand these trajectories in 3D by extracting the Z-position encoded in the particle images by classification against a reference library of defocused patterns at known Z-levels (Fig. 1). We leverage a deep learning model for Z-position prediction (SI Appendix, Fig. S4) and compare its performance against normalized cross-correlation on experimental data.

### Evaluating accuracy and precision

**Fig. 2:**
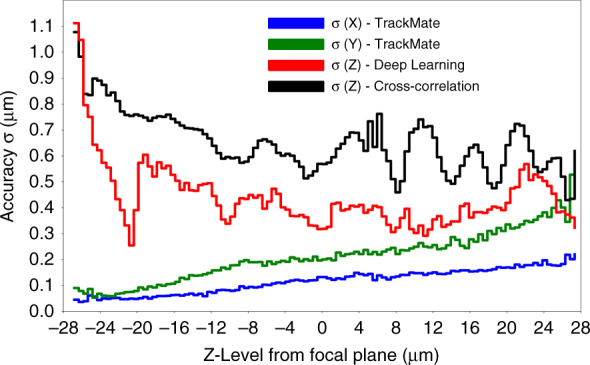
Method accuracy using synthetic images. Accuracy (σ) in X, Y, and Z is reported as the RMSE between predicted and actual coordinates across the *n* = 110 Z-levels comprising the working range. The deep learning model (in red) shows an improved σ (0.41 µm) over the cross-correlation algorithm (in black, 0.63 µm)

Accuracy (denoted *σ*) represents the agreement between predicted Z-positions and ground-truth labels, and precision (denoted *E*_*prec*_) the extent of the low-amplitude variation around the average Z-position along an in-plane displacement. We quantified the accuracy of the XY localization *via* TrackMate using synthetic images (SI Appendix, Fig. S5), and of the Z-localization *via* cross-correlation and our deep learning model. We report the root mean square error (RMSE) between the predicted and ground-truth label values as the measure of *σ* (Fig. 2, see Material and Methods). XY *σ* is minimum (0.04 µm in X, 0.05 µm in Y) near the negative extreme of the Z-range and increases roughly linearly to a maximum (0.20 µm in X, 0.40 µm in Y) at the positive extreme. Overall, the median XY *σ* is 0.12 µm in X and 0.21 µm in Y. We attribute this discrepancy in X and Y to a slight distortion of the defocus pattern of particles, which becomes more apparent with increasing Z (SI Appendix, Fig. S6). For Z-localization, the prediction *σ* for the cross-correlation and deep learning strategies fluctuate across the median for most of the Z-range, only increasing near the negative extreme at Z < −25 µm. This result indicates that the Z-range (h) is adequately sized but is approaching the limits of the optical system at the lower end of the range. For the cross-correlation method, the median *σ* is 0.63 µm with a normalized value σ/h = 0.012 (h = 54 µm) in agreement with previously reported performance^14^. However, in contrast to a previous report^23^, our deep learning model provides a higher accuracy than cross-correlation with a value of 0.41 µm with σ/h = 0.0075. The normalized value σ/h may be improved in our case by expanding the Z range in the positive direction because that region exhibits a low *σ*. Decreasing the step size is unlikely to improve σ/h because the step size is comparable to the median *σ* values and thus the resolution.

To estimate precision, we quantified the Z-position of particles using experimental data, for which no ground truth is available but whose trajectories are predictable. We recorded high-speed videos of a Poiseuille flow through a straight rectangular channel. Pathlines are expected to be monotonous with little change in defocus pattern across their linear trajectory. Thus, variation in Z-position across each track reflects only the imprecision of the prediction method. We inject seed particles suspended at 0.25 mg/ml in a 45/55 (% v/v) solution of water in glycerol to match the refractive indices of the single-phase and PDMS. The microfluidic channel has a 90 µm × 35 µm (width × height) rectangular cross-section. After predicting the Z-position of particles in each frame, we calculate the precision *E*_*prec*_ as the median RMSE along each pathline fitted against a 1-D Z(X,Y) linear regression model to account for a slight tilt of the system. *E*_*prec*_ for both methods are similar, with 0.14 µm for the cross-correlation and 0.17 µm for the deep learning prediction, with non-parametric interquartile ranges of 0.06 µm (SI Appendix, Fig. S7). The Y-*E*_*prec*_ is 0.12 µm. The Z-*E*_*prec*_ of our method is comparable to this value, which underscores its capability to measure full-3D pathlines at high resolution.

### Re-labeling training images is critical for the deep learning model

We considered a dataset with a tilt around the Y-axis to assess the impact of re-labeling image particles. In this instance, the particle Z-positions in the reference image for the 0 µm level vary linearly from −2 µm to +2 µm as determined by cross-correlation (SI Appendix, Fig. S8). We quantify the impact of re-labeling on the precision measurement in a horizontal Poiseuille flow. In the absence of re-labeling, the deep learning model provides a lower average value for the pathlines slope (−0.0012 µm/µm *vs*. −0.0036 µm/µm), while the *E*_*prec*_ is almost twice as large as with the cross-correlation (0.26 µm *vs*. 0.14 µm). After re-labeling and retraining the deep learning model, the *E*_*prec*_ for the deep learning model decreases from 0.26 µm to 0.17 µm, and the median slope is identical to the cross-correlation case (0.0036 µm/µm). The cross-correlation method is less sensitive to biased labeling because it compares particle images to the median image of each Z-level, which eliminates the most extreme deviations from the image motifs. It is noteworthy that visual inspection cannot detect a 4 µm tilt across the field of view. Our results highlight a critical aspect of deep learning applications to defocusing µPTV using experimental (rather than synthetic^23^) training sets: labeling errors or bias must be systematically evaluated and corrected. Even slight perturbations or misalignment of the microfluidic device relative to the focal plane can cause labeling errors that significantly impact the performance of the deep learning model.

### Channel Step Experiment

**Fig. 3:**
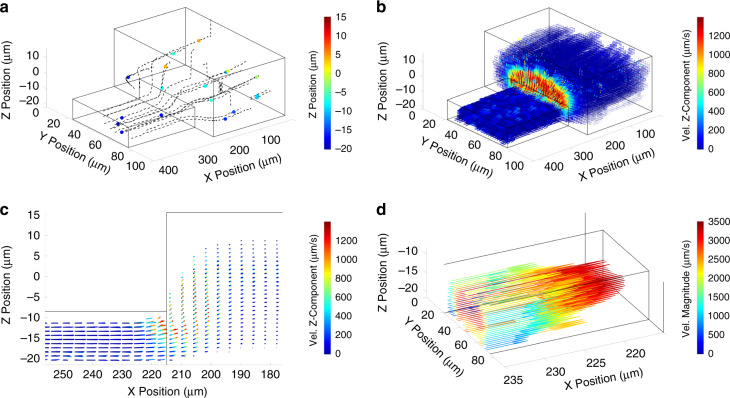
Flow maps at a channel step using the cross-correlation algorithm. Vectors sourced from *n* = 79,814 spots across 701 pathlines with a median length of 121 spots. **a** Isometric view of 14 selected individual pathlines. **b** Isometric view of the lattice-averaged 3D velocity vector field using a 4 µm × 2 µm × 1 µm element size. **c** XZ planar side view of the velocity field. Vector length codes for the velocity magnitude and color codes for the value of its Z-component. **d** Parabolic velocity profile characteristic of a Poiseuille flow observed upstream of the channel step

We characterized a single-phase flow at a channel step where a 45 µm wide and 13 µm deep cross-section expands to a 90 µm wide and 35 µm deep cross-section (Fig. 3). We injected a 0.52 mg/ml bead suspension at 5 µL/hr and captured 701 pathlines with a median length of 121 spots for a total spot count of 79,814 from a single 8300 frame video taken at 300fps and 1920 × 1080 resolution.

Both classification methods generate very similar pathlines and yield very few gross errors (SI Appendix, Fig. S9). The scarcity of near-wall information is conspicuous as near-wall flow is difficult to capture. Similar to all seed particle-based methods, flow velocities cannot be measured within one particle radius (1.5 µm here) from the walls. Near-wall particles are rare because of the low seed density inherent to defocusing-based µPTV. In contrast to other defocusing-based µPTV approaches relying on fluorescence microscopy, the use of brightfield enables the precise location of channel walls or other stationary features.

3D trajectories illustrate the topography of hydrodynamic flows, but we can also extract the velocity fields that provide critical information, such as hydrodynamic forces. We calculated the instantaneous velocity vectors from between-frame particle displacements and then lattice-averaged them. We filtered out unphysical vectors greater than a *w*_*max*_ of 1300 µm/sec (see Material and Methods), which corresponds to the fastest out-of-plane motion just downstream of the step expansion (Fig. 3b, c). Both classification methods generate very few unphysical vectors: 20 for the cross-correlation method and 4 the deep learning model, out of a total of 79,112 instantaneous vectors. We lattice-averaged the 416 µm × 93 µm × 43 µm (X,Y,Z) measurement volume with a 4 µm × 2 µm × 1 µm element size and 50% overlap. This lattice yielded a median count of 10 vectors for non-empty elements. Upstream of the step expansion, the flow is unidirectional, and the parabolic velocity profile typical of Poiseuille flow is visible (Fig. 3d). About 10 µm before the expansion, the flow begins diverging upward in Z and symmetrically outward in Y. The flow expansion continues for several tens of µm downstream of the step as the streamlines expand to fill the greater channel volume. The maximum out-of-plane velocity is observed immediately downstream of the expansion before asymptotically returning to zero in the downstream flow. The upward flow expansion continues very gradually at about 40 µm downstream of the step.

### Continuity Error Analysis

In absence of ground truth, we assess the physical validity of the velocity field of the channel step by evaluating its deviation from the conservation of mass, or continuity equation, that should be zero for a non-compressible flow (*SI Appendix* Continuity Error Analysis)^24^. We evaluated the scalar η (continuity error parameter) field for the channel step dataset across a range of lattice element sizes. We considered a subvolume centered on the step expansion (178 µm< x < 255 µm in Fig. 3c) because this region contains most of the out-of-plane flow and is thus the most sensitive to Z-prediction. The median value across the scalar field, η^~^, typically varies from 0 (perfect continuity) to 1 (uncorrelated velocity components). η ~ is smaller than 0.5 for all element sizes, except 1 µm × 1 µm × 1 µm (Table 1). η ~ is maximum for the smallest element size, 1 µm × 1 µm × 1 µm, and then declines with increasing element sizes to a minimum at 4 µm × 4 µm × 4 µm, before increasing slightly again at 6 µm × 6 µm × 6 µm. These results follow the expected evolution of η ~ as a function of the element size. Smaller element sizes are more sensitive to noise or errors because they contain fewer vectors. This is confirmed for the 1 µm × 1 µm × 1 µm element size by looking at its distribution (*SI Appendix* Continuity Error Analysis and Fig. S10). In addition, continuity is affected when the element size becomes comparable to the precision of particle location, which increases the chance of assigning a vector to the wrong lattice element. Thus, η ~ is expected to be greater for small element sizes, to decline with increasing element sizes, until the element size encompasses flow vectors with true different directions and generates discretization errors. Classification by cross-correlation and deep learning also produced very similar results (Table 1).

**Table 1:** Variation of $\tilde \eta$ with lattice element size

Lattice Element Size	$\tilde \eta$ (Cross-correlation)	$\tilde \eta$ (Deep learning)
4 µm x 2 µm x 1 µm	0.45	0.50
1 µm x 1 µm x 1 µm	0.88	0.90
2 µm x 2 µm x 2 µm	0.30	0.29
4 µm x 4 µm x 4 µm	0.14	0.14
6 µm x 6 µm x 6 µm	0.17	0.16

In addition, we investigated the effect of rectangular lattice elements. In the channel step case, the resolution of the out-of-plane (Z) and lateral (Y) motion near the step expansion is more critical than the resolution along the streamwise (X) direction. A 4 µm × 2 µm × 1 µm lattice element (used size in Fig. 3) enables to resolve the flow to 1 µm in Z without exhibiting a loss in continuity. The result is also confirmed by plotting the scatterplots of Δu/Δx vs. –(Δv/Δy + Δw/Δz) (*SI Appendix* Continuity Error Analysis and Fig. S11). This result suggests that users can adjust the lattice element to increase the resolution of the velocity field along a specific axis. Overall, the continuity analysis of the velocity field of the channel step confirms that it follows the conservation of mass.

### Computation cost analysis

We compared the computational costs of the cross-correlation and the deep learning-based Z-prediction algorithms, by analyzing a set of 2681 particle image time-stacks with a median track length of 53 frames. The dataset contains particle images extracted from 4 high-speed videos aggregated from 3 experiments performed at the same flow rate (25 µL/hr), with different focal plane heights and local flow characteristics. The deep learning model ran on a GPU (NVIDIA GeForce RTX 2070 SUPER, 7.79GB), while the cross-correlation algorithm was run on a Windows 10 PC with Intel i5-4570S (2.9 GHz) 4-core processor and 16 GB RAM.

Training of the neural network took 4572 s (about 76 min). Classification by the deep learning model was 2 orders of magnitude faster than classification by cross-correlation: the median Z-prediction time per stack across 2681 time-stacks was 0.049 s via the deep learning model and 20.6 s via cross-correlation (SI Appendix, Fig. S12). The cost of the deep learning model training is also one order of magnitude lower than the classification time by cross-correlation in our example. This difference will only grow larger with larger dataset, for which the speed advantage of the deep learning model increases. It is important to note that, in our case, the deep learning model can use GPU scaling while the cross-correlation implementation used is CPU limited. These results underscore that deep learning models are well suited for applications involving a series of experiments, such as design optimization of a complex microfluidic geometry and high-resolution flow mapping.

### Single-phase flow induced by displacement structures

**Fig. 4:**
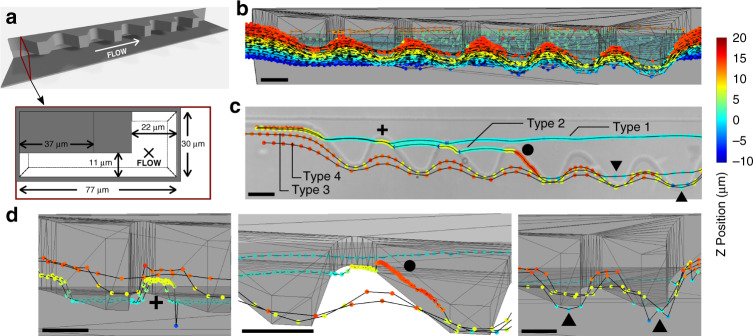
Mapping the flow generated by displacement structures. **a** 3D view (*Top*) and cross-section (*Bottom*) of the displacement structures. They include jagged overhangs that displace large particles towards the opposite wall and a particle exclusion section that diverts only fluid to maintain large particles displaced. **b** Isometric view of *n* = 22,151 spots across 262 pathlines that highlight wave-like flow between jagged structures. **c** Top view projection of 4 stereotypical trajectories overlaid onto a micrograph of the displacement structures. **d** Magnified views of these 4 pathlines that underline the dramatic changes in depth of the Type 1 (*Left*, cross), Type 2 (*Middle*, circle), and Types 3 and 4 trajectories (*Right*, triangles) caused by the structures. Scalebars represent 25 µm

Displacement structures enable capture of single cells or particles with near-perfect efficiency by shifting their center of mass within the streamlines that flow through chambers^22^. They consist of 19 μm thick overhangs in a 30 μm deep channel designed to exclude large particles and act as a conduit to divert flow underneath (Fig. 4a). It has been suggested that particles are displaced due to the helicoidal shape of the flow^22^; however, the flow pattern induced by those structures has not been detailed experimentally.

We injected a solution of seed particles at 0.42 mg/ml at 5 µl/hr close to the overhangs using a 20 µl/hr co-flow. The flow map, generated with the cross-correlation algorithm, shows four trajectory types depending on the initial proximity of the seed particles to the wall. Particles close to the wall tend to remain underneath the overhangs (Type 1, cyan, and dark blue trajectories in Fig. 4b) or get displaced upward and outward after interaction with a tooth (Type 2). Interestingly, seed particles that follow Type 1 and 2 trajectories start at the same distance from the wall but at different depths. Seed particles further away from the wall are displaced right away towards the tip of the first tooth (Type 3 and 4) but can slip under one of the following teeth and even divert back towards the wall (Type 3). All particles exhibit vertical wavy trajectories when interacting with the teeth (Fig. 4c, d Type 1 to 4). Interestingly, we can enumerate the number of specific pathlines to confirm how the displacement structures manipulate the fluid and displace large particles to the opposite wall (Table 2). The proportion of pathlines that flow under the structure (Type 1) is higher than that of pathlines diverted outwards (Type 2). Also, the proportion of pathlines that slip under the tips and towards the wall (Type 3) is much lower than that of pathlines that remain at the structure tips (Type 4). These proportions challenge the direct effect of a helicoidal flow pattern but instead support the role of flow diversion in the streamline jumping effect. Indeed, our data support a mechanism where the structure constrains large particles to a portion of the channel section while the flow diverted underneath the structure acts as a co-flow that maintain particles in their new positions. 3D trajectories of seed particles highlight the complexity of the flow generated by the displacement structures and tease out the underlying mechanism that drives particles to change streamlines.

**Table 2:** Proportion of each trajectory type across displacement structures

Type 1	Type 2	Type 3	Type 4
79%	3.4%	15.6%	1.9%

We further verified that the deep learning model generated similar pathlines (data not shown). Finally, we changed the position of the focal plane, from below to above the bottom of the structures, to confirm that it has no incidence on the flow maps (data not shown).

### Two-phase droplet flow in a straight rectangular channel

We validate our method by mapping the internal flow of microfluidic droplets, a case that is notoriously challenging because of the optical distortion due to the interface. We generated 1.1 nL droplets that flowed into a channel with a 120 µm wide and 38 µm deep rectangular cross-section. We collected data on two movies containing 54 and 34 droplets with mean seed densities of 3.7 and 2.6 particles *per* droplet. We produced two datasets to assess the impact of data loss near the interface. The first one, called the Auto dataset, uses only spots detected by TrackMate. The second, called the Corrected dataset, includes missed spots (false negatives) that we added manually. TrackMate’s detection (true positive) rate for the Auto dataset is 91% and 96% for the two movies.

**Fig. 5:**
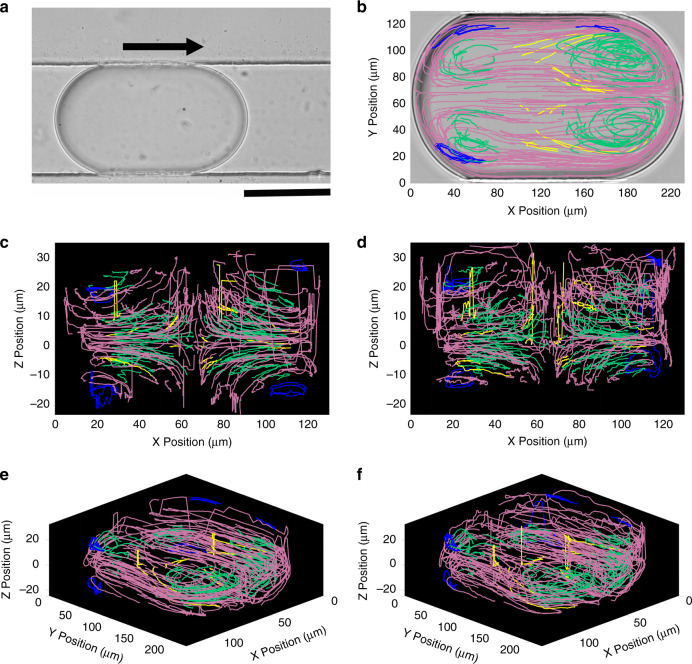
Droplets in a straight channel (pathlines) from the Corrected dataset. Data represent *n* = 23,521 spots across 305 pathlines with a median length of 79 spots (the Auto dataset contains 22,172 spots across 370 pathlines with a median length of 76 spots). Recirculation flows are manually colored to distinguish topological patterns: large recirculation pathlines in pink; corner vortices in green; transition between red and green flows in yellow; small vortices in vertical extremes of endcaps in blue. **a** A 1.1 nL droplet with sparse seed particles flowing in a straight channel of 120 μm wide and 38 μm deep cross-section. The scale bar represents 100 μm. **b** Top-down projection of pathlines onto the XY plane. **c, d** Front end view of 3D pathlines obtained by the cross-correlation algorithm **c** and the deep learning model **d**. **e, f** Isometric views of 3D pathlines generated with the cross-correlation algorithm **e** and the deep learning model **f**.

The flow maps generated illustrate the topological characteristics of the internal flow of droplets (Fig. 5, Corrected dataset). The recirculation flow exhibits mid-planes of symmetry both vertically (XZ) and horizontally (XY), as shown by the end-on view of the 3D pathlines in Fig. 5c, d. Those planes divide the flow into 4 quadrants spanning the length of the droplet. We manually colored the pathlines to highlight features of the flow structure: pink pathlines depict recirculation flow around the perimeter of each quadrant, green pathlines depict local vortices in the front and rear of each quadrant, and yellow pathlines represent transitions between the red and green patterns. In microfluidic droplets, the internal flow topology is strongly affected by the capillary number Ca^25,26^ and channel width/height aspect ratio^27^, and topological regimes can be delineated by critical Ca values that are in turn dependent on the inner-to-outer viscosity ratio λ^25^. The topology revealed here reflects a low-Ca regime described previously^26,27^. This is expected given the transition trends described by Jakiela et al.^25^ and the low Ca (6.9 × 10^−4^) and λ (0.67) values in this case. In rectangular channels, the continuous phase typically overtakes droplets through gutters located between the interface and the channel corners^25^. The shear induced by the higher velocity gutter flows propagates through the interface and drives the recirculation flow within droplets. Even at low *Ca*, the viscous shear stress exerted by the gutter flow on the droplet slightly deforms the front endcap. This effect increases its curvature and surface area, which results in a greater transfer of viscous energy across the front endcap and larger local vortices at the droplet front (Fig. 5a, b). Intriguingly, the blue pathlines depict small local vortices found in the vertical extremes of the droplet near the junction between the endcaps and the channel walls (see SI Appendix, Movie S1 for a sequence that reveals one of the blue vortices). These vortices are located at the extremities of the gutters (Fig. 5e, f) and may be driven by the splitting of the continuous phase into gutter flows. Notably, the thickness of those vortices is in the order of a few μm, and to our knowledge, they have not been described with other techniques. Our approach allows to describe the flow map in minute detail.

**Fig. 6:**
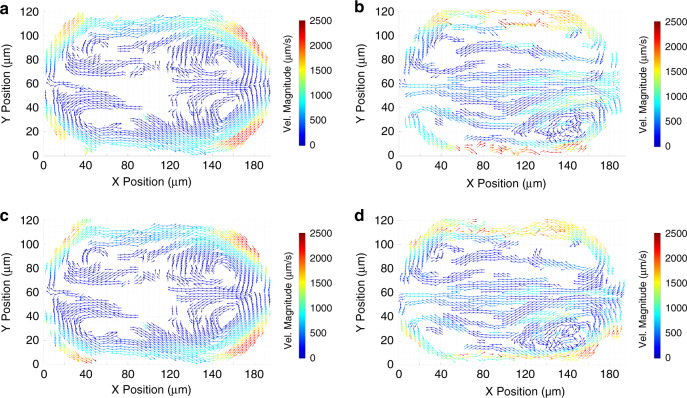
Droplets in a straight channel (velocity field). XY Planar views of the lattice averaged velocity vector field with an element size of 4 µm × 2 µm × 4 µm (ΔX, ΔY, ΔZ). Average vectors are color-coded for magnitude. Maps were generated from a total of *n* = 23,216 instantaneous velocity vectors from the Corrected dataset. **a**, **b** Velocity fields generated by the cross-correlation algorithm at Z = 1.2 µm and Z = 13.2 µm respectively. **c**, **d** Velocity fields generated by the deep learning model at Z = 1.2 µm and Z = 13.2 µm respectively.

Both classification methods generate Z-prediction errors, reflected in single-error spikes and “error plateaus” (sustained errors over several consecutive positions). These errors are more common near the droplet interface (Fig. 5c–f) and at a higher rate for the deep learning model (Fig. 5c and e and d and f). They are due to confusing motifs in the particle images, such as the interface or nearby particles, for which the model is not trained. We applied a 5th-order median filter along each pathline sequence of Z-coordinates to smooth out these errors (*SI Appendix*, Materials and Methods). We calculate the instantaneous velocity vectors and lattice-average them using a 4 µm × 2 µm × 4 µm element with a 50% overlap, which results in a median of at least seven instantaneous vectors *per* non-empty element (Fig. 6). Those velocity vectors can additionally be filtered to remove unphysical values. In this case, we established a physical *w*_*max*_ limit of 1000 µm/sec (*SI Appendix*, Materials and Methods). Downstream vortices appear larger than the upstream vortices and are covered by a greater density of vectors, revealing the axial front-rear asymmetry of the local recirculation flow within each quadrant. We notice the flow of the continuous phase through the gutters that is indicated by the faster near-interface downstream flow in the 13.2 µm plane compared to the 1.2 µm plane.

Interestingly, we do not observe backward flow near the channel walls in the 1.2 µm plane. Typically present in lower aspect ratio channels (1.0^25^ and 1.5^26^), the backward flow is indicative of the viscous drag due to the oil film between the droplet and the channel wall. Our experiment used a much higher aspect ratio channel (3.2), and more closely reflects the µPIV results of Li et al.^27^ that also (in a 2.5 aspect ratio channel) showed only forward-directed flow near the channel walls at the mid-plane in the low Ca regime. Increasing the channel aspect ratio reduces the film area and the viscous energy transfer across the side interfaces, thinning or eliminating the band of backward-directed internal flow adjacent to the interface^27^. Our results further underscore that the channel aspect ratio is a critical parameter determining the topology of the internal flow of microfluidic droplets.

Finally, we compare the Auto (21,777 vectors) and Corrected datasets (23,216 vectors) to evaluate the relevance of the latter by quantifying the number of unphysical vectors in both sets and after applying a 5th order median filter to pathlines (*SI Appendix*, Table S2). Overall, the Corrected dataset does not improve quantitatively because the correction adds only a few percent of velocity vectors. Those numbers do not significantly affect the vector density and hence the element size or spatial resolution. The foreign image features challenge both the spot detection by TrackMate and Z prediction algorithms; however, the Corrected dataset addresses only the detection step but not the classification step. Yet, the Corrected dataset partially restores the loss of velocity vectors near the interface. Vectors in those regions are disproportionally filtered out due to the presence of the interface in images (a few % of the vectors are lost near the interface compared to a few 0.1% in the center of droplets). All considered, the 5th-order median filter has the most significant impact on recovering data near the interface compared to the effect of the Corrected dataset that requires manual input.

Using our technique, we generate a highly detailed 3D map of the recirculation topology inside microfluidic droplets. Our results are consistent with the general patterns of recirculation described previously using µPIV^4,5,9^. Coincidently, the fully-3D pathline-level mapping permits the detection of novel features such as the narrow corner vortices that cannot be easily detected with µPIV because of their shallow thicknesses. The spatial resolution of the velocity field is fully tunable and can be adjusted by collecting additional data.

### Two-phase droplet flow in a semi-circular rectangular channel

**Fig. 7:**
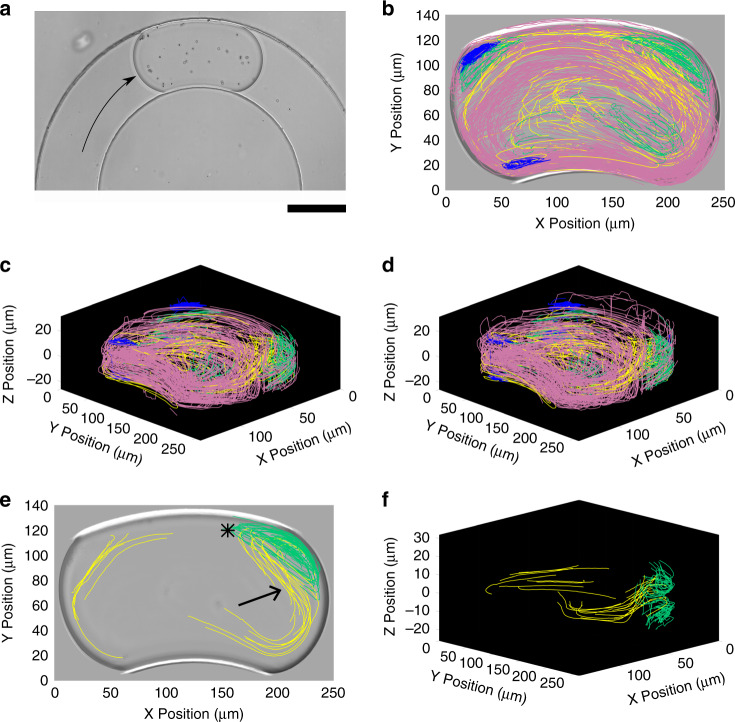
Droplets in a curved channel (pathlines) from the Corrected dataset. Data represent *n* = 25,738 spots across 1,270 pathlines with a median length of 17 spots (the Auto dataset contains 23,225 spots across 1,365 pathlines with a median length of 12 spots). The coloring scheme is similar to Fig. 5. **a** A 1.1 nL droplet with sparse seed particles flowing into a curved channel of radii 190 μm and 310 μm, and a 120 μm wide and 38 μm deep cross-section. The scale bar represents 100 µm. **b** Top-down projection of pathlines. **c**, **d** Isometric views of pathlines generated by the cross-correlation algorithm **c** and deep learning model **d**. **e** Top-down projection of pathlines highlighting the folding shape of the transition pathlines (yellow) and their relationship with the top vortex (green). **f** Isometric view of the transition pathlines that highlights their 3D folding shape. Data shown are generated with the cross-correlation approach.

We use our method to study the mixing effect of a curved channel within microfluidic droplets. We generated 1.1 nL droplets that flowed in a rectangular channel with a 120 µm wide and 38 µm deep cross-section that describes an arc section (Fig. 7a). We recorded the motion of 40 droplets with a mean density of 17.8 single seed particles. TrackMate’s detection rate was 78.1% in the Auto dataset.

We manually colored the pathlines to highlight the flow structure using the previous nomenclature after applying a 5^th^-order median filter (Fig. 7b–f, Corrected dataset). Consistent with the straight channel results, more errors are visible in the deep learning than cross-correlation dataset, especially near the droplet interface. Here the higher seed density likely increases the frequency at which interface and foreign-particle motifs are observed in particle images, increasing the overall error rate (Fig. 7c, d). Small narrow vortices (blue) are also present at the extremities of the gutter at the droplet rear (SI Appendix, Movie S2). We observe an asymmetry between the principal inner and outer main recirculation vortices (pink) (Fig. 7b). Other topological features recall those observed in the straight channel case with some variations. The local recirculation vortices (green) are seen in both the front and rear of the droplet, but the downstream vortices are larger and more irregular. The channel curvature induces a radial asymmetry due to a difference in the shear induced by the gutter flows. The wider inner vortex is driven by a higher flowrate than the outer vortex because the continuous phase splits between gutters of different lengths and thus hydrodynamic resistances in this case. Indeed, interfacial energy predominates over viscous, centrifugal, and inertial effects because of the low Capillary, Dean, and Weber numbers ($Ca \approx 1.10^{ - 3}$, $De \approx 0.01$, $We \approx 2.10^{ - 5}$). Minimal interfacial deformation is expected, and the relative hydrodynamic resistances of the gutters depend only on their relative lengths. We estimate the relative resistance ratio to be about 1.6 based on the 56° arc angle of the droplet (*SI Appendix*, Materials and Methods).

Importantly, the yellow pathlines emphasize the transition between the well-defined red and green vortices and illustrate the enhanced in-droplet mixing observed in curved channel (Fig. 7b–f). Here, tracer particles travel from one vortex to another along the yellow pathlines (SI Appendix, Movie S3). They curve backward from the downstream lower corner (arrow in Fig. 7e) before sharply turning back (asterisk in Fig. 7e) into the downstream upper green vortex. Those trajectories have a pronounced Z-displacement (Fig. 7f) and appear to cross pink pathlines (Fig. 7b). This result clearly illustrates the ability of our method to reveal critical information about flow structures.

**Fig. 8:**
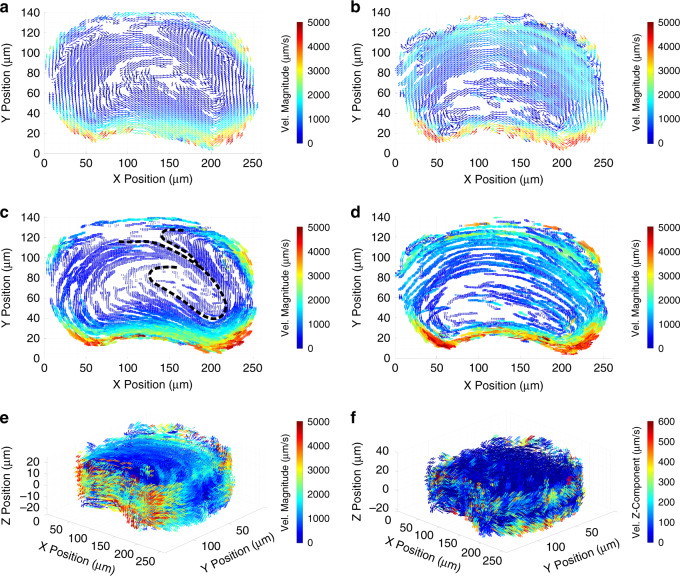
Droplets in a curved channel (velocity field). Lattice averaged velocity vector fields processed with the deep learning model (see SI Appendix, Fig. S8 for cross-correlation results). Maps were generated from a total of *n* = 24,226 instantaneous velocity vectors from the Corrected dataset. **a**, **b** XY planar fields at **a** Z = 1.2 µm and **b** Z = 13.2 µm with an element size of 4 µm × 2 µm × 4 µm dimensions. Color codes for the velocity vector magnitude. **c**, **d** Same data as panels **a** and **b** with a smaller element size of 2 µm × 1 µm × 4 µm dimension. The data better highlight the flow folding close to the midplane (dashed lines in in panel **c**). **e**, **f** Isometric views of the 3D velocity field where color codes for the vector magnitude and codes for velocity out-of-plane (*w*) component respectively.

We lattice-average the vector field using both a 4 µm × 2 µm × 4 µm lattice element and a 2 µm × 1 µm × 4 µm lattice element with 50% overlap (Fig. 8). These element sizes result in a median of 7 and 2 instantaneous vectors *per* non-empty element, respectively (*SI Appendix*, Table S3). The cross-correlation and deep learning results are similar (SI Appendix, Fig. S8). Critically, the flow structure illustrates the mixing mechanism close to the midplane. The inner and outer vortices communicate *via* a region between 150 µm < X < 200 µm (Fig. 8a and c). Flow from the inner vortex is diverted upward to the outer section of the droplet and joins the local recirculation vortex shown in the upper right. The downstream highest velocity flow near the droplet interface completes the folding effect. The bulk of the droplet interior is sparsely defined, indicating a low-velocity, low-flow region inside the wide inner vortex. The folding effect is more apparent in the map generated with the smaller lattice element (Fig. 8c vs. 8a). Closer to the top interface, no mixing-inducing folding is evident (Fig. 8b and d). We observe the signature of the gutter flow near the interface in the top plane and its higher velocity next to the inner surface (Fig. 8e). By selecting the Z-component of velocity vectors, we can underscore that most out-of-plane velocities occur primarily in the droplet endcaps (Fig. 8f). Taken together, those findings support our hypothesis that the asymmetry seen in the internal recirculation flow is due to an asymmetry of the gutter flow and not a centrifugal effect.

Our droplet data showcase the strength of our method by revealing several novel features of the topology of their recirculation flow. Droplets flowing in a straight channel develop small recirculation vortices at their corners. Also, droplets in a semi-circular channel exhibit a centrifugal-like effect despite flowing at a low Dean number. Our high-resolution 3D flow map identifies the mixing pattern induced by the channel curvature in three dimensions. The folding pattern could not be observed with PIV techniques that tend to average movement in XY and lack high resolution in Z.

## Discussion

Our results demonstrate that we can generate a 3D map of experimental microflows at micron-scale resolution using a brightfield microscope equipped with a low numerical aperture objective, a simple set-up that makes it easily accessible to the scientific community. It delivers sub-micron precision and accuracy in three dimensions. Additionally, our strategy provides a greater temporal resolution than fluorescence^28,29^, phase contrast^14^, or pinhole-aperture^30–32^ approaches that limit light collection. Data presented in this report were recorded at up to 5 mm/s, in contrast to other methods that perform both multi-frame particle tracking and velocimetry at lower velocities^32,33^. Only an expensive optical set-up can provide higher temporal resolution with fluorescence microscopy^17^. Our approach also precisely locates channel walls and other stationary features.

The accessibility of our method is furthered by the use of the open-source tracking algorithm TrackMate, available as a standard Fiji plugin. Our data indicate that TrackMate can provide sub-micron localization of seed particles and efficiently link particle positions between frames. However, the algorithm can be challenged by foreign structures or overlapping particles and could benefit from integrating more advanced detection and anti-collision algorithms^34–37^.

Using a reference library to determine the Z-position of seed particles is essentially an image classification problem. For that reason, we developed a deep learning model and compared its performance to a cross-correlation method. Overall, both classification methods yielded similar performances and results when applied to experimental data. Our work highlights that assessing and correcting for labeling bias in the training set is critical when using a deep learning model. The relative performance of the deep learning model could be attributed to the structure of the motifs to classify. The defocusing patterns possess limited features and change very gradually between classes. At this point, it is unclear whether deep learning will surpass cross-correlation methods in classifying Z-positions for experimental data. Cross-correlation methods are robust and do not require as many reference images; however, the presence of foreign objects in images challenge both methods. Critically, deep learning models can use GPU scaling and are adapted to process large amount of data at high-speed even after taking into account the initial collection of images to create the training library. The fact that recent plugins, such as DeepImageJ^38^ (not used in this manuscript), enables the implementation of trained deep learning models in ImageJ further underscore the modularity and accessibility of our workflow. Deep learning could also be used to generate artificial libraries to classify non-symmetrical objects, such as mammalian cells whose defocus patterns cannot be approximated using microspheres like bacteria^14^. Conversely, ray tracing algorithms can be used to generate libraries of non-symmetrical objects^39^. Alternatively, cell images taken at a few discrete Z levels can be interpolated to alleviate the labor-intensive task of collecting enough library images^18^. Finally, it remains to be seen how a library and a trained deep learning model could be shared between laboratories.

We validate our method on two highly relevant microfluidic examples. First, our data highlight the flow structure induced by displacement structures and elucidate how it efficiently shifts large particles across streamlines. Second, our high-resolution 3D flow maps of microfluidic droplets reveal novel recirculation structures and folding patterns that could not be previously observed with typical flow mapping techniques like PIV. These examples demonstrate the capabilities of our method to generate true 3D flow maps at high spatial and temporal resolution. Compared to PIV methods, our strategy maps microflows in three-dimension and at high spatial and temporal resolution. High depth-resolution in PIV requires elaborate illumination schemes and expensive high NA objectives. PIV also tends to spatially average information as it cross-correlates textural information created by seed particles between frames. In contrast, our data reveal the power of observing individual pathlines to uncover flow patterns. For instance, we observed crossing streamlines in displacement structures and uncovered critical details of the folding pattern of droplet internal flow in curved channels. Revealing those novel patterns hinges on the ability to track individual trajectories of seed particles. Our method will support the design and development of novel microfluidic structures to address the growing demand for manipulating samples at the microscale.

## Materials and methods

For a complete description of the Materials and Methods, see Supplementary Information (SI) Materials and Methods.

### Experimental set-up

An inverted motorized microscope (Eclipse Ti-E, Nikon, Tokyo, Japan) controlled by NIS Elements software (Nikon), a 20x/0.45NA objective (Plan Fluor ELWD, Nikon), a fiber-optic LED illuminator (SugarCUBE, Ushio, Tokyo, Japan) and a high-speed camera with a 1920 × 1080 sensor array and a 0.32 µm × 0.32 µm pixel size (Q-MIZE HD v2, AOS Technologies AG, Baden, Switzerland) are used to capture videos. We perform experiments with the aperture diaphragm open at 75% and the field aperture fully open. The correction ring is adjusted to 0.2 mm despite using 1 mm thick glass slides to induce asymmetry in the Point Spread Function and eliminate ambiguity in the Z-position of particles about the focal plane. One mL glass syringes (Gastight #1001, Hamilton, Reno, NV, USA) actuated by a low-pulsation syringe pump (NEMESYS 290 N, Cetoni GmbH, Korbußen, Germany) are used to inject solutions into microfluidic devices *via* a PEEK tubing (0.254 mm ID, 0.787 mm OD, Zeus, Orangeburg, SC, USA).

### Microfluidic designs and microfabrication

Microfluidic circuits are designed with 2D CAD software (AutoCAD, Autodesk, San Rafael, CA, USA) (*SI Appendix*, File S1), and printed onto transparency masks (CAD/ART Services, Bend, OR, USA). PDMS devices are fabricated using soft lithography^40^. For a complete description of the microfluidic designs and their microfabrication, see SI Materials and Methods.

### Seed particle solutions

3 µm polystyrene beads (#100223-10, Corpuscular, Cold Spring, NY, USA), (25 mg/mL) are used for the experiments. The particle solution is first diluted with water and then mixed with glycerol in a 45/55 (% v/v) ratio to match the refractive index of PDMS. The particle suspension is vortexed for 30 seconds and sonicated for 30 min to break up particle aggregates. Addition of Tween-80 (Fisher Scientific, Fair Lawn, NJ, USA) up to 0.8% (v/v) substantially reduces particle aggregation. The final suspension is vortexed for 30 seconds. For the droplet experiments, the continuous phase is a 31.9/68.1 (% v/v) mixture of perfluorohexyloctane (F6H8, Apollo Scientific, Stockport, UK) in mineral oil (Sigma, St. Louis, MO, USA), also to match the refractive index of PDMS. The dispersed phase remains a solution of water in glycerol at 45/55 (% v/v) without Tween-80. For description of seed particle selection and refractive index matching, see SI Materials and Methods.

### TrackMate

TrackMate^21^ involves two steps: (1) spots are detected within frames at a sub-pixel resolution (Fig. 1a), and (2) spots are linked across frames to create particle pathlines (“tracks”). A Laplacian-of-Gaussian (LoG) filter is applied by TrackMate to a movie frame-stack and spots are detected as local maxima^41^. The generated tracks may contain false positives (erroneous spots), false negatives (missed spots), and/or linking errors (crossed tracks). The TrackScheme tool is used to correct only false positives and linking errors. The resulting dataset is referred to as the Auto dataset. In droplet experiments, missed spots are more often located near the droplet interface. A “Corrected” dataset is generated by manually placing spots in the Auto dataset to recover missed spots (false negatives). This adds a spot to a pathline but does not modify the raw image of the bead located at that spot. For each track, a time-stack is created by extracting 32 × 32 pixel images centered on each spot of the trajectory. The Z-position of each spot is then predicted by image classification against the reference stacks *via* cross-correlation or the deep learning model.

### Generation of reference stacks

The same source images are used to create the cross-correlation reference stack and the deep learning model’s training set. They consist of 32 × 32 pixel particle images that are extracted from video frames recorded at incremental distances from the focal plane by moving the objective during recording with the high-speed camera. A script in NIS Elements (Nikon) is executed to move the objective down to the lower limit of the Z-range and then up to the upper limit in discrete increments equal to the step size along the Z-axis. The step size is corrected for the refractive index mismatch between the objective immersion medium (air) and the working fluid^42^:

1 $$Objective\,Step\,Size = \frac{{Desired\,Z - step\,Size}}{{n_{wf}/n_{air}}}$$

The reference stack covers a 54 µm Z-range with a 0.5 µm step size (*N*_*steps*_ = 110). We determined the focal plane (Z = 0 µm) using the maximum Brenner gradient, and the other Z-levels are labeled based on the number of steps away from the focal plane. For a complete description of the generation of reference stacks, see SI Materials and Methods.

### Relabeling reference images

Labeling of reference images using the focal plane assumes that the fields of view are perfectly horizontal. This is not the case in practice, and particle images from the same FOV may not represent the same Z-position, especially for Z-steps as small as 0.5 μm and a large FOV. We assess for slide tilt and re-label the reference images to correct for this effect; for a complete description, see SI Materials and Methods.

### Cross-correlation algorithm

For a complete description of the cross-correlation algorithm, see SI Materials and Methods.

### Deep learning model

A deep learning model is trained on labeled particle images to predict the Z-position. The prediction of the Z-position is formulated as a regression analysis. In contrast to a classification task that selects from discrete classification labels, a regression task can produce continuous outcomes with enhanced interpretability. The regression model is built on a Resnet-50^43^ convolutional neural network architecture with a depth of 50 layers and is pre-trained on the ImageNet^44^ dataset. Instead of connecting the output of one convolution block to the next block, Resnet-50 includes new skip connections that connect the original input to the output of the current block for improved performance. Such new skip connections enable the free flow of gradients and thereby help reduce the vanishing gradient problem^43^. Resnet-50 is expanded into a model for regression analysis by adding a flatten and regression head layer (Fig. S4). The machine learning library Autokeras is used to train our deep learning model^45^. All reference images are partitioned into the training, validation, and testing sets with a ratio of 64:16:20.

### Error calculations (accuracy σ and precision *E*_*prec*_)

We calculate the accuracy metric of our technique, σ, as the root mean square error (RMSE) between measured values along the in-plane (X,Y) and out-of-plane (Z) axes and the corresponding ground-truth values:

$$\sigma = \sqrt {\frac{{\mathop {\sum }\nolimits_{i = 1}^N a_{predicted} - a_{ground - truth}}}{N}}$$

Where *a*_*predicted*_ is the coordinate value predicted *via* TrackMate (for in-plane X,Y predictions) or *via* either cross-correlation or deep learning (for predictions along the Z-axis), and *N* is the number of independent predictions at a given ground-truth value.

We calculate the precision metric, *E*_*prec*_, similarly but we replace the known ground-truth value with a value predicted by a linear regression model for a given pathline. Here, *a*_*model*_ = Z(X,Y) for out-of-plane precision or *a*_*model*_ = Y(X,Z) for in-plane precision and *L* is the pathline length (number of spots/frames):

$$E_{prec} = \sqrt {\frac{{\mathop {\sum }\nolimits_{i = 1}^L a_{predicted} - a_{\bmod el}}}{L}}$$

### Filtering unphysical velocity vectors

Sharp jumps in Z-position resulting from gross errors typically produce unphysically large velocity values. Instantaneous velocity vectors whose magnitude is beyond an unphysical limit (*w*_*max*_) are filtered out. Determination of *w*_*max*_ is described in the SI Materials and Methods.

### 3D Visualization with a web browser based graphic user interface (GUI)

All data presented in this paper can be explored with a web-based GUI at: https://www.stonybrook.edu/commcms/defocusing_3D_mapping/index.html. For a detailed description of this interface, see the SI Materials and Methods.

### Single phase (displacement structures)

A particle suspension, diluted to 0.42 mg/ml of 45/55 (% v/v) water in glycerol, is injected at 5 µl/hr into a microfluidic channel with overhanging displacement structures. The particles are focused against the structures using a 20 µl/hr co-flow of a bead-free solution of the same composition. The illumination is adjusted to match the mean grey value of the reference stacks and the objective position so that particles flow both above and below the focal plane. A single high-speed video is recorded at 1920 × 1080 resolution, with 8300 frames at 300 frames/sec with a 150 µs exposure. For description of post-processing and TrackMate results, see SI Materials and Methods.

### Internal droplet flow

The flow field is mapped inside microfluidic droplets flowing in a straight channel and along a semi-circular curve. A particle suspension, diluted to 1.04 mg/ml of 45/55 (% v/v) water in glycerol, is used as the dispersed phase. Flow rates of 10 µl/hr and 40 µl/hr are used for the dispersed and continuous phases, respectively. The illumination is adjusted to match the mean grey value of the reference stacks and the objective position so that particles flow both above and below the focal plane. For the straight channel experiments, two high-speed videos are recorded at 1920 × 1080 resolution, each with 8,300 frames taken at 500 frames/sec with a 150 µs exposure. The videos contain 54 and 34 drops, respectively. For the curved channel experiment, a single 1920 × 1080 resolution video is recorded with 8300 frames taken at 300 frames/sec with a 150 µs exposure, capturing 98 droplets in total, of which we post-processed only the first 40. Droplets are sufficiently spaced such that only a single droplet is captured within the FOV at any given time. For description of post-processing and TrackMate results, see SI Materials and Methods.

### Coordinate transformations of droplet data

The challenge of transforming the TrackMate data to the droplet’s internal reference frame is different for the straight and curved channel cases. In the straight channel case, stacks of images centered and cropped around every droplet are compiled to subtract the droplet’s bulk motion implicitly. In the curved channel case, transforming 2D TrackMate data to the reference frame of the droplet requires compensating for the angular displacement of the droplet along the curve. That displacement is computed by substracting the droplet arc displacement to the individual bead displacement as a function of their radial position. For complete descriptions of these coordinate transformations, see SI Materials and Methods.

### Dimensionless numbers

For the droplet experiments, the Reynolds number *Re* is calculated as ${\it{Re}} = \rho V_dD_h/\mu _d$ where $\rho ,\overline {V_d} ,D_h,$ and *μ*_*c*_ are the fluid density, droplet velocity, hydraulic diameter of the channel cross-section, and dynamic viscosity of the continuous phase respectively. *Re* = 0.013 and 0.022 for the straight and curved channel cases respectively. The relative magnitude of viscous, inertial, and buoyancy forces are compared to the interfacial tension *ϒ* by calculating the Capillary number ($Ca = \mu _cV_d/\gamma$), Weber number ($We = \rho V_d^2D_h/\gamma$), and Bond number ($Bo = \Delta \rho gD_h^2/\gamma$). $Ca = 6.9 \times 10^{ - 4}$ and $1.2 \times 10^{ - 3}$ and $We = 9 \,\times 10^{ - 6}$ and $2.4 \,\times 10^{ - 5}$ for the straight and curved droplet experiments respectively, and $Bo = 1.6 \,\times 10^{ - 4}$ for both cases. Thus, interfacial tension is expected to dominate the droplet dynamics with minimal interfacial deformation, and buoyancy and fluid inertial forces can be neglected. For the curved channel experiment, the Dean number $\kappa = \sqrt {w/2R} Re$ is calculated to be 0.011 ≪ 1, where *w* is the channel width and *R* is the mean arc radius. Below *κ* ~ 1, secondary Dean flow is not expected to be a significant component of the continuous phase flow or the internal droplet recirculation.

## Supplementary information

Revised supplemental information file

Movie S1

Movie S2

Movie S3

File_S1

File S2

File S3

File S4

File S5

File S6

File S7

File S8

File S9

File S10

File S11

File S12

## Data Availability

Full pathline-level datasets generated and described in this paper are available as Supplementary Files S2–S12. These datasets can also be navigated with a Graphic User Interface at https://www.stonybrook.edu/commcms/defocusing_3D_mapping/index.html.

## Code availability

All scripts used in this paper are freely available on Github (https://github.com/brouzes-lab-sbu-bme/3D_defocus_tracking).
