# Supplementary material for: Widely accessible method for 3D microflow mapping at high spatial and temporal resolutions
Source: Microsyst Nanoeng. 2022 Jul 1;8:72. doi: 10.1038/s41378-022-00404-z (PMC9246883; doi:10.1038/s41378-022-00404-z)
Supplement: Supplementary file 1 — Revised supplemental information file [file 41378_2022_404_MOESM1_ESM.docx]

**Supplementary Information for**

# Widely accessible method for 3D microflow mapping at high spatial and temporal resolutions

Evan Lammertse^1^, Nikhil Koditala^5^, Martin Sauzade^1^, Hongxiao Li^5^, Qiang Li^5^, Luc Anis^1^, Jun Kong^5^, and Eric Brouzes^1,2,3,4^

^1^ Department of Biomedical Engineering, Stony Brook University, Stony Brook, NY, USA 11794.; ^2^ Laufer Center for Physical and Quantitative Biology, Stony Brook University, Stony Brook, NY, USA 11794; ^3^ Cancer Center, Stony Brook School of Medicine, Stony Brook, NY, USA 11794; ^4^ Institute for Engineering Driven Medicine, Stony Brook University, Stony Brook, NY, USA 11794; ^5^ Department of Mathematics and Statistics, Department of Computer Science, Georgia State University, Atlanta, GA, USA 30302

*Corresponding Author: Eric Brouzes

Corresponding Author’s Email: eric.brouzes@stonybrook.edu

Corresponding Author’s Office Phone: (631)-632-1852

Corresponding Author’s Fax: (631)-632-8577

Author Emails:

Evan Lammertse: evan.lammertse@stonybrook.edu

Nikhil Koditala: nkoditala1@student.gsu.edu

Martin Sauzade: martin.sauzade@gmail.com

Hongxiao Li: hli35@gsu.edu

Qiang Li: qli23@student.gsu.edu

Luc Anis: luc.anis@stonybrook.edu

Jun Kong: jkong@gsu.edu

**This PDF file includes:**

Supplementary text

SI Materials and Methods

Curved Channel Gutter Flow Ratio

Continuity Error Analysis

Seed Density Analysis

Figures S1 to S13

Tables S1 to S4

Legends for Movies S1 to S3

Legends for Files S1 to S12

SI References

**Other supplementary materials for this manuscript include the following:**

Movies S1 to S3

Files S1 to S12

### **Supplementary Text**

### SI Materials and Methods

**Microfluidic designs.** All cross-sections are rectangular. The single phase (displacement structures) circuit is a 2-layer design. The first layer is a full-width rectangular cross section while the second layer contains the displacement structures overlayed onto the channel. The total channel height is 30 µm with the underside of the structures 11 µm above the channel bottom. The channel width is 77 µm. The smallest structure extends 37 µm from the wall. Subsequent structures extend further outward to 55 µm at the final of six structures (Fig. 3a). The straight and curved channel droplet experiments use the same design that contains three distinct parts: 1) a 180 µm square flow-focusing junction to generate droplets, 2) a 1.73 mm long straight section, 3) an arc section with an inner wall radius of 190 µm and outer wall radius of 310 µm. The cross-section is constant, 120 µm wide and 38 µm deep, throughout the straight and arc portions.

### **Microfabrication.** The SU-8 negative photoresist (Kayaku Advanced Materials, Westborough, MA, USA) is patterned onto 3” silicon wafers using a UV mask aligner (500W UV illuminator, Newport, Irvine, CA). PGMEA (Kayaku Advanced Materials) is used to develop structures. A previously developed protocol^1^ is used to generate a double-layer mold for the channel step experiments. Molds are silanized overnight by vapor deposition with a fluorinated silane compound (1H,1H,2H,2H-Perfluorooctyltrichlorosilane, Gelest, Morrisville, PA) to facilitate PDMS release. Molds are mounted in an acrylic jig to ensure the uniform thickness of the PDMS slabs. PDMS (Sylgard 184 Silicone Elastomer Kit, Dow Corning, Midland, MI) slabs are molded at a 10:1 (polymer:crosslinker) weight ratio, after degassing under vacuum and curing for 1 hour at 80ºC. To create ports, the PDMS slabs are punched with a biopsy tool (Syneo, West Palm Beach, FL, USA). The PDMS surfaces are cleaned with tape (Scotch 810 Magic Tape, 3M, Saint Paul, MN, USA). Slabs are bonded to a 2” x3” glass slide (Corning, Inc., Corning, NY, USA) spin-coated with a 150 µm thick PDMS layer after activation with an oxygen plasma (PDC-32G, Harrick Plasma, Ithaca, NY). This procedure ensures identical surface properties of the four walls of the channels. For single-phase experiments, native PDMS is used. For droplet experiments, channels are coated with a 5% (v/v) mixture of heptadecafluoro-1,1,2,2-tetrahydrodecyl)trichlorosilane (Gelest) in HFE-7500 (3M) for 5 minutes right after bonding; the coating solution is flushed out with pure HFE-7500 oil.

**Seed particle selection.** 1.02 µm and 3 µm diameter cross-linked polystyrene microbeads (Corpuscular #100211-10 and 100223-10, Corpuscular, Cold Spring, NY), 1.02 µm diameter silica microbeads (#140210-10, Corpuscular), 2 µm glass microbeads (#9002, Thermo-Fisher Scientific, Waltham, MA, USA), and 1 µm magnetic microbeads (#S1419S, New England Biolabs, Ipswich, MA, USA) are tested as tracer particles. The smallest diameter beads are used to optimize the optical set-up (Fig. S1) and 3 µm polystyrene beads (25 mg/mL) are used for the experiments.

**Refractive index matching.** We estimate the refractive index of the 45/55 (% v/v) water in glycerol solution used as the dispered phase using the Arago-Biot formula:

$$n_{mix}=n_{1}\times\theta_{1}+n_{2}\times\theta_{2} (1)$$

where $n_{i}$ and $\theta_{i}$ are the refractive index and volume fraction of the pure components, respectively, to be $n_{aq}=1.409$, which nearly matches the refractive index of PDMS (1.412).

Mapping the 3D internal flow of microfluidic droplets requires minimizing the refractive index mismatch between the two phases to minimize optical distortion by the droplet curvature. This distortion is more significant near the interface where horizontal and vertical recirculating flows converge and bifurcate^2^, resulting in most out-of-plane flow occurrence. The continuous phase is a 31.9/68.1 (% v/v) mixture of perfluorohexyloctane (F6H8, Apollo Scientific, Stockport, UK) in mineral oil (Sigma, St. Louis, MO, USA). Using the Arago-Biot formula, the refractive index of the oil mixture is estimated to be $n_{oil}=1.427$, which approximates the refractive indices of the aqueous phase ($n_{aq}=1.409$) and of the PDMS ($n_{PDMS}=1.412$) and allows for clear imaging of tracer particles even near the interface.

**Interfacial tension measurements.** The interfacial tension of this fluid system is measured using the pendent drop technique^3^. Briefly, a syringe containing the continuous phase is mounted in a vertically oriented syringe pump (NE-300, New Era Pump Systems, Farmingdale, NY, USA). The syringe is manually dispensed into a cuvette filled with the dispersed phased and images of the pendent drop at equilibrium are captured using a USB microscope (#1061, Adafruit, New York, NY, USA) with AMCap software^4^. After calibrating the pixel size in µm, “Pendent Drop”^5^, a free ImageJ plugin, is used to extract the drop profile and calculate the interfacial tension. This technique is then validated with a fluid system of known interfacial tension (1X PBS in HFE-7500 with 2% w/w PFPE-PEG surfactant)^6^. The dynamic viscosity of each phase is measured using a Cannon-Feske routine type viscometer (No. 100, 945D, CANNON Instrument Company, State College, PA, USA) to first measure the kinematic viscosity which is then corrected for the density. The fluid properties are listed in Table S4.

**Generation of reference stacks.** A particle suspension, diluted to 0.2 mg/mL of 45/55 (% v/v) water in glycerol, is injected into a large (3 mm x 8.3 mm) PDMS microchannel. Particles are randomly dispersed across the field of view (FOV) after sedimentation. To ensure an adequately extensive training set for the deep learning model, a 10:1 ratio between the number of training images *per* step ($N_{source}$) and the number of steps ($N_{steps}$) is targeted. This rule entails 1,100 source particle images *per* Z-level. In our study, 1,064 training images *per* Z-level sourced from 14 FOVs are used, containing an average of 76 particles each. As a preprocessing step, stacks are divided by a particle-free background image of the FOV, converted to 8-bit greyscale, and then multiplied by a constant to restore the initial mean grey value. For each frame, TrackMate^7^ is used to determine particles X,Y coordinates and extract 32x32 pixel images centered on them.

To extract the Z-level of each frame, the average diffraction pattern of beads is plotted across the whole recording. Discontinuities are identified that correspond to an incremental change in focus. Particle images in each video frame are stacked and median-projected to generate a 32x32x$N_{frames}$ “median stack” spanning the entire Z-range. A 5x5 convolution filter (see Strategy section) is applied to highlight the diffraction rings. The stack is then XZ-resliced to observe the evolution of the particle diffraction patterns across the Z-range. In each row of the image, bright pixels indicate the position of the diffraction rings, and discontinuities in the bright vertical lines indicate the stepping of the objective between frames (Fig. S2). Those discontinuities are used to group frames of the same objective position. Using the maximum Brenner gradient, the focal plane (Z = 0 µm) is determined, and the other Z-levels are labeled based on the number of steps away from the focal plane. After labeling source images, cross-correlation reference stacks are generated by creating a 32x32x$N_{steps}$ stack of the median particle images at each Z-level, from all FOVs. The deep learning training set consists of 32x32x $N_{source}$ stacks for each Z-level, containing images of all unique isolated particles at that Z-level.

**Relabeling reference images.** To assess the tilt of FOVs, the Z-position of the particle images is established from the nominal Z-level of 0 µm using the cross-correlation algorithm. A linear regression is then fitted to the horizontal X and vertical Y particle coordinates against their Z-position. Similar X and Y *vs.* Z regression models are also fitted at each of the other Z-levels in the training set to check for similar tilt. Overall, no tilt is seen in Y *vs.* Z, but X *vs.* Z shows noticeable tilt that requires correction (Fig. S7). The Z-labels of particle images are corrected by re-binning the particle images as a function of their location in the FOV. The median linear model parameters (slope and intercept offset from the nominal Z-level) are first calculated across all Z-levels. Training images are relabeled with a new Z-label if the median linear model predicts the Z-position within +/- 0.25 µm of the label. This criterion is chosen because we use a Z-step size of 0.5 µm.

### **Cross-correlation algorithm.** To predict a particle’s Z-position by cross-correlation, the test and reference stacks are multiplied by themselves, the 32-bit result is converted to 8-bit, and the following 5x5 convolution filter is applied:

$$\left[ \begin{matrix} \begin{matrix} -0.5 & -0.5 & -0.5 & -0.5 & -0.5 \\ -0.5 & -0.5 & -0.5 & -0.5 & -0.5 \\ -0.5 & -0.5 & 12 & -0.5 & -0.5 \\ -0.5 & -0.5 & -0.5 & -0.5 & -0.5 \\ -0.5 & -0.5 & -0.5 & -0.5 & -0.5 \end{matrix} \end{matrix} \right]$$

The Pearson’s correlation coefficient^8^ *r* between a test image $x$ and a reference image $y$ is used:

$$r= \frac{\sum_{i=1}^{N} (x_{i}-x_{m})(y_{i}-y_{m})}{\sqrt{\sum_{i=1}^{N} {(x_{i}-x_{m})}^{2}}\sqrt{\sum_{i=1}^{N} {(y_{i}-y_{m})}^{2}}} (3)$$

Where $N$ is the total number of pixels in each image, $x_{i}$ and $y_{i}$are the intensity values of the $i^{th}$ pixels in the test and reference images, $x_{m}$ and $y_{m}$are the mean intensity values of the test and reference images. Eq. 3 is equivalent to:

$$r=\frac{{CI}_{peak}-Nx_{m}y_{m}}{N\sigma_{x}\sigma_{y}} (4)$$

where ${CI}_{peak}$ is the peak intensity near the center of the cross-correlation image $CI= x*y$ and $\sigma_{x}$ and $\sigma_{y}$ are the standard deviations of the intensity of the test and reference images. The spatial domain calculations *per* equation 3 are cumbersome; however, equation 4 allows for a much faster calculation of $r$ using ImageJ’s “FD Math…” function to generate $CI$ in a single function call. The equivalency between cross-correlation in the spatial domain with complex conjugate multiplication in the frequency domain is used. The Z coordinate of each TrackMate spot is determined using an ImageJ macro to calculate a correlation coefficient $r$ against every slice (Z-level) in the reference stack *via* eq. 4. The spot’s Z-position can be predicted as the discrete Z-level with maximum $r$ value. However, the Z-position is further refined to continuous output by determining the location of the maximum of a 2nd-order polynomial that fits the 5 points centered on the maximum cross-correlation value.

**3D data output to MATLAB.** An ImageJ macro is used to append the predicted Z-positions to TrackMate-generated .csv files, extending the data from 2D to 3D. The .csv files containing 3D data are loaded into TrackMate and the “Export tracks to XML file” action is executed to generate MATLAB-compatible .xml files. MATLAB is used to process the 3D data at the pathline level, calculate instantaneous and lattice-averaged velocity fields, and visualize the data in 3D. TrackMate .xml files are imported into MATLAB *via* the importTrackMateTracks.m function.This function returns a 1-D array of cells that contain the successive X,Y,Z,T coordinates of the tracked particle in a single TrackMate track. The track data is preprocessed by: 1) truncating Z-positions outside the limits of the Z-range to the nearest limit; 2) determining the minimum and maximum X,Y,Z coordinates of each track, from which global X,Y,Z limits of the 3D field volume are determined; and 3) applying an optional median filter along each track’s array of Z-positions to filter out gross errors. Selecting a filter order/kernel size requires balancing the filter's power to smooth out error plateaus and the loss of spatial resolution associated with large kernel sizes. To strike this balance, we tried odd kernel sizes from 3 to 11 and calculated the number of error velocity vectors lost to the $w_{max}$ filter (see Filtering unphysical velocity vectors) in each case. The largest number of velocity vectors filtered out by the $w_{max}$ threshold occurs in the absence of a median filter (noisiest data). We selected a 5th order median filter because 84.5% of unphysical error vectors are recovered with this kernel size, while an 11th order filter recovers only an additional 7.6% compared to the case without median filer, for the droplet datasets in the straight channel. Similar results were obtained for the curved channel case, while no median filter was necessary for single-phase data.

The instantaneous velocity vectors are calculated and placed at the midpoint along the displacement vector. Vector fields are stored as 6xn double arrays in which columns represent the vector X,Y,Z-positions and corresponding U,V,W velocity components, and n is the total number of vectors. The instantaneous vectors are then lattice-averaged over the 3D field volume with a user-input element size and 50% overlap between adjacent elements. For each element, the average of all instantaneous vectors inside the element is calculated and placed at the centroid of the element volume. The Quiver 3D function^9^ is then used to visualize the lattice averaged field in 3D, with vectors colored according to magnitude or w velocity component.

### **Determination of** $\boldsymbol{w}_{\boldsymbol{max}}$ **for error vector filtering.** To determine $w_{max}$, the number of filtered vectors are plotted against a wide range of possible $w_{max}$ values, with decreasing values from 2,000 µm/sec to 100 µm/sec in 100 µm/sec increments. $w_{max}$ is selected to maximize the number of erroneous vectors and minimize the number of physical vectors filtered. If $w_{max}$ becomes too low, the number of filtered vectors sharply increases indicating that many physical vectors are being removed. $w_{max}$ is selected one increment just above the first large spike in filtered vectors. Erroneous vectors can also be filtered based on unphysical locations (e.g., outside of the channel walls). For both droplet experiments, it is set to 1,000 µm/sec.

### **3D Visualization with a web browser based graphic user interface (GUI).** The web GUI is built using Vite (<https://vitejs.dev/>), a JavaScript bundler that allows for using Node Package Manager modules. The interface is created using Three.JS (<https://threejs.org/>), a JavaScript library for WebGL that allows rendering 3D graphics in a browser. The canvas containing the 3D environment is added using JavaScript. HTML is used for overlays such as the sidebar. The complete set of pathlines and the “jet” colormap are exported from MATLAB to .json files using the “jsonencode” function. The codebase is divided into three parts: an index.html file, a main.js file, and supporting assets (JSON files, STL files, images, etc.). In the main.js file, the “lineGroup” variable is used to contain all the parts of the scene that must be recreated when changing between datasets and/or colormap schemes. The “addSet” function is used to add a dataset to the lineGroup. A nested loop over each pathline is used to create a Vector3 object for each coordinate point and sequentially order the coordinates in an array. Data are mapped to the 256 levels “jet” colormap. The GUI can display three types of data: 1) Z-position; 2) velocity; and 3) velocity Z-component. Velocities are forward calculated as the Euclidean distance to the next coordinate divided by the time interval between frames.

### **Synthetic images for accuracy measurement.** A stack of synthetic images containing particle images at known locations are generated to evaluate the XY tracking accuracy (Fig. S4). Each slice of the cross-correlation reference library is pasted at the nodes of a 15x9 array, equally spaced 128 pixels apart, onto a background of an empty PDMS channel. Gaussian noise is added to the median projections to match the mean and standard deviation of the noise of the source images.

### **Single phase (displacement structures) post-processing and TrackMate results.** The frames are imported to ImageJ/Fiji as a time-stack and cropped. The frames are preprocessed similar to the reference stacks: image stacks are divided by the stack median (a particle-free background image), converted to 8-bit greyscale, and then multiplied by a correction factor to restore the pre-division mean grey value. 262 TrackMate tracks with a median track length of 69 spots are extracted for a total of 22,151 spots. The video contains an average number of single particles (excluding particle aggregates) *per* frame $N_{p}$of 3.0 (95% confidence: 2.7-3.3). TrackMate detects an average number of spots *per* frame $N_{p}$’ of 2.7, for a recall parameter ø = $N_{p}$’ ⁄$N_{p}$ of 90%. This equates to a mean particle image density $N_{s}$ of 0.062 and a mean measured particle image density $N_{s}'$ of 0.057. The mean particle image density $N_{s}$ and the mean measured particle image density $N_{s}'$ of are defined as:

$$N_{s}= N_{p}\left( \frac{A_{p}}{A_{i}} \right), N_{s}'= N_{p}'\left( \frac{A_{p}}{A_{i}} \right) (5)$$

Where $A_{p}$ is the typical particle image area and $A_{i}$ is the image area within the channel walls. $A_{p}$ is calculated as the median area inside the largest unbroken diffraction ring across each slice of the cross-correlation reference stack.

**Internal droplet flow post-processing and TrackMate results.** Frames are preprocessed similarly to the single-phase experiment before extracting isolated droplet stacks to transform the coordinate system implicitly (see below, “Coordinate Transformations of Droplet Data”). Our methods are then applied to the isolated droplet stacks. The first and second videos of droplet flow through a straight microchannel contain 54 and 34 droplets with mean seed densities $N_{p}$ of 3.7 +/- 2.0 and 2.6 +/- 1.6 (mean +/- SD) single particles *per* droplet, for mean particle image densities $N_{s}$ of 0.016 and 0.011, respectively. The videos yield a combined total of 7,885 frames of droplet motion, after removing empty frames and those with only partial droplets. The Auto and Corrected datasets (see Strategy section) respectively contain a total of 373 and 305 tracks, with median track lengths of 76 and 79 spots for total spot counts of 22,172 and 23,521. The measurement volume is determined from the mean droplet length and the channel width and height to be 212 µm x 122 µm x 38 µm. In the Auto datasets, TrackMate detects on average ${N_{p}}^{'}$= 3.4 +/- 1.7 and 2.5 +/- 1.4 spots *per* frame from the first and second videos, respectively, resulting in measured particle image densities ${N_{s}}^{'}$ of 0.014 and 0.011 and recall φ of 91% and 96%. In the Corrected datasets, ${N_{p}}^{'}$= 3.6 +/- 1.8 and 2.6 +/- 1.4 from the first and second videos, respectively, while ${N_{s}}^{'}$= 0.015 and 0.011, for detection rates of 96% and 100%.

For the curved channel experiment, the raw video is imported as a time-stack in ImageJ and our standard preprocessing algorithm is applied. The stack is manually split into sub-stacks to isolate each droplet. These 1,920x1,080 full-FOV droplet stacks are ran through TrackMate and a coordinate transformation is applied to the resulting 2D pathline data to map data in the reference frame of the droplet.

### **Coordinate transformations of droplet data.** In the straight channel case, the movie stack is binarized to produce black droplet borders against a white background to identify each droplet in the movie. The stack is vertically cropped to a band of pixel rows at the center of the channel, 20 rows wide, the median of this band for each frame is calculated, and then ImageJ’s “Reslice...” function is used to generate a 1,920x8,300 pixel X-T resliced image. Each row of the resliced image represents a movie frame, and the white/black boundaries in each row indicate the horizontal positions of the droplet front and rear in that frame. ImageJ’s “Analyze Particles...” function is used to detect these boundaries and generate a list of ROI points locating droplet boundaries in each frame. For each droplet’s travel across the FOV, these boundary points are used to extract droplet images and combine them into a single time stack. Droplet displacement is thus implicitly subtracted between frames, and TrackMate can measure 2D particle trajectories within the droplet’s reference frame.

In the curved channel case, the arc displacement component is the product of the angular displacement of the droplet between frames and the radial position of the bead in the first frame from the center of the arc channel. The 90° (vertical) position along the arc is used as the reference droplet. The following are determined: 1) the location of the center of the semi-circular channel arc; 2) the location of the droplet centroids in each frame; 3) the angular displacement of droplets between frames; 4) the angle between each droplet and the reference droplet centered at the vertical; 5) the distance between beads and the center of the semi-circular channel arc; and 6) the droplet mean included arc angle.

The following procedure is used to determine the center location of the semi-circular channel: 1) a 5-pixel radius minimum filter is applied to a background image of the channel walls before it is binarized; 2) a Hough circle transform is used to extract the center location of the four concentric semi-circular features in the image (the outer and inner sides of the outer and inner channel walls); and 3) the center location of the semi-circular path is estimated as the component-wise mean of those four center locations.

Next, the droplet centroid locations in each frame are determined. The ImageJ “Find Edges” filter is applied to each droplet stack to highlight droplet boundaries and channel walls. A droplet-free background image is subtracted from the stack to highlight bright droplet boundaries against a dark background. Three successive smoothing operations are applied before binarizing the images. Six successive 3x3 dilation filters, followed by six 3x3 erosion filters are applied to restore the original droplet area. The “Fill Holes” operation is used to generate white droplet areas against a black background. Finally, the “Analyze Particles” function is used to extract the centroid location of each droplet. The arc displacement component is subtracted from the particle displacement vector for each particle-frame pair to transform the particle coordinates from TrackMate into the droplet’s reference frame.

The arc displacement is estimated by multiplying the particle’s distance to the center of the semi-circular channel in the first frame by the angular displacement of the droplet between frames. The displacement vector is then transformed to the frame of the reference droplet at the 90° position based on the droplet position in the first frame. The 2D coordinate data is normalized to the mean droplet size to account for slight variations in droplet size when consolidating data from different droplets. The droplet size is defined by the arc angle of the droplet, which is measured using the following procedure: 1) a semi-circular arc is fitted to the series of droplet centroids; 2) Intersection points between the droplet boundaries and this arc are recorded; and 3) the arc angle of the droplet is formed by the intersection points and the
center of channel arc. Particle coordinates at the reference droplet are then arc-shifted based on the ratio of the arc angle of the droplet and the arc angle of the mean droplet.

Curved Channel Gutter Flow Ratio

For an average droplet arc angle of 56 degrees and inner and outer radii of 190 µm and 310 µm, the resistance ratio becomes $\frac{R_{outer}}{R_{inner}}=1.63$ for a continuous phase flow rate ratio $\frac{Q_{outer}}{Q_{inner}}=0.61$. This ratio roughly matches the relative cross-sectional widths of the inner and outer red vortices in Fig. 6b.

Continuity Error Analysis

We assess the validity of the velocity field by evaluating its deviation from the conservation of mass, or continuity equation, that should be zero for a non-compressible flow. We thus compute the continuity error parameter η over the lattice volume using the following formulation^10^:

$$\eta=\frac{\left( \frac{\partial u}{\partial x}+\frac{\partial v}{\partial y}+\frac{\partial w}{\partial z} \right)}{\left( \frac{\partial u}{\partial x} \right)^{2}+\left( \frac{\partial v}{\partial y} \right)^{2}+\left( \frac{\partial w}{\partial z} \right)^{2}}=1+\frac{2\left( \frac{\partial u}{\partial x}\frac{\partial v}{\partial y}+\frac{\partial u}{\partial x}\frac{\partial w}{\partial z}+\frac{\partial v}{\partial y}\frac{\partial w}{\partial z} \right)}{\left( \frac{\partial u}{\partial x} \right)^{2}+\left( \frac{\partial v}{\partial y} \right)^{2}+\left( \frac{\partial w}{\partial z} \right)^{2}}$$

$\eta$ can vary between 0, when the continuity equation is perfectly satisfied, to 3, for the special case of $\frac{\partial u}{\partial x}=\frac{\partial v}{\partial y}=\frac{\partial w}{\partial z}$. $\eta$ can be estimated from experimental velocity fields using central differences:

$$\eta_{i,j,k}=\frac{\left( \frac{u_{i+1,j,k}-u_{i-1,j,k}}{x_{i+1,j,k}-x_{i-1,j,k}}+\frac{v_{ij+1,k}-v_{i,j+1,k}}{y_{i,j+1,k}-y_{i,j+1,k}}+\frac{w_{i,j,k+1}-w_{i,j,k-1}}{z_{i,j,k+1}-z_{i,j,k-1}} \right)}{\left( \frac{u_{i+1,j,k}-u_{i-1,j,k}}{x_{i+1,j,k}-x_{i-1,j,k}} \right)^{2}+\left( \frac{v_{ij+1,k}-v_{i,j+1,k}}{y_{i,j+1,k}-y_{i,j+1,k}} \right)^{2}+\left( \frac{w_{i,j,k+1}-w_{i,j,k-1}}{z_{i,j,k+1}-z_{i,j,k-1}} \right)^{2}}$$

Alternatively, we used a one-sided difference equation for elements with only one non-zero neighbor element in a given coordinate direction. The median value, $\tilde{\eta}$, typically varies from 0 to 1, and the higher the value the more uncorrelated $u, v$, and $w$ are^10^.

To further investigate the dependence of the continuity error parameter η to the element size, we estimated its density distribution on the (0,1) interval using the MATLAB’s ksdensity() function (Fig. S10). A distribution more skewed towards the 0 value indicates a better agreement with the continuity equation. We notice that the distributions of η for all the elements size display a peak 0 < $\eta$ < 0.1. However, the distribution of $\eta$ for the smallest element size exhibits an additional peak close to 1, which corresponds to a random distribution of velocity vectors. The evolution of the distributions with increasing element sizes indicates a tightening of the distributions towards the 0 value before broadening again for the 6mm^3^ element size. Both the evolution of the values and distribution of $\eta$ are in agreement with previous PIV investigations^10^, which underscores the validity of our method.

In addition, we investigated the effect of rectangular lattice elements. In the channel step case, the resolution of the out-of-plane (Z) and lateral (Y) motion near the step expansion is more critical than the resolution along the streamwise (X) direction. A 4µm x 2µm x 1µm lattice element (used size in Fig. S9) enables to resolve the flow to 1µm in Z without exhibiting a loss in continuity (absence of peak close to 1 in Fig. S11). This result suggests that users can adjust the lattice element to increase the resolution of the velocity field along a specific axis.

Alternatively, we visualized the continuity error by plotting the scatterplots of Δu/Δx vs. –(Δv/Δy + Δw/Δz) (Fig. S11). In these plots, the 45° line represents perfect concordance with the continuity equation, and a greater spread about this line represents greater continuity error^10^. The data spread about the 45° line increases with decreasing element size, reflecting the results in Fig. S10 and Table 1 that suggest an increase of continuity error with decreasing element sizes. Interestingly, the spread about the 45° line for the lattice elements 2µm x 2µm x 2µm and 4µm x 2µm x 1µm are very similar, which confirms that resolution can be sacrificed along one axis to gain resolution in another (X and Z in this case, respectively) while maintaining a similar level of continuity error.

Seed Density Analysis

A “dropped spot” error is a failure to accurately locate a spot (usually during an overlap event), either because it is present but off-center, or failed to be detected. A “particle linking error” is a failure to maintain a consistent association between a TrackMate track object and the same particle.

To estimate the optimal seed density, we injected seed solutions at four particle image densities (0.003, 0.007, 0.009 and 0.028) into a rectangular microchannel with a 98µm width and a 44µm height cross-section. For each suspension, we captured 2000 frames of high-speed video at 1920x1080 resolution and 300fps with a 150 µs shutter time. We used TrackMate to automatically generate 2D tracks from these videos and reported the percentage of dropped spots and particle linking errors for each condition (Table S5).

To estimate the optimal seed density, we injected seed solutions at four particle image densities (0.003, 0.007, 0.009 and 0.028) into a rectangular microchannel with a 98µm width and a 44µm height cross-section. For each suspension, we captured 2000 frames of high-speed video at 1920x1080 resolution and 300fps with a 150 µs shutter time. We used TrackMate to automatically generate 2D tracks from these videos and reported the percentage of dropped spots and particle linking errors for each condition (Table S5).

The dropped spot rate increases only modestly over an eightfold increase in particle image density (Fig. S13a), from 0.8% at $N_{s}= .003$ to 2.6% at $N_{s}= .028$. The linking error rate increases more dramatically, from an average of 0.08 linking errors/particle at $N_{s}= .003$ to nearly 2 errors per particle at $N_{s}= .028$. The efficiency of TrackMate’s spot detection remains high as the seed density increases. However, more manual effort is required to correct dropped spots and linking errors if the application demands unbroken pathlines. These data support the need for a more robust particle linking algorithm to increase the data density for pathline analysis.

**Figures S1-S14**

**Figure S1.**

**Fig. S1** **XZ Sections showing various tracer particle defocus patterns.** Patterns are distinguished by material, size, and percent of the aperture diaphragm opening. We selected polystyrene particles with a 70% diaphragm opening as optimizing pattern asymmetry, cone angle, and depth of field. We used a 3 µm particle size instead of the 1 µm size shown here to increase the particle image area and pixel data available for neural network model training and cross-correlation (Fig. S2a).

**Figure** **S2.**

**Fig. S2** **Cross-sections of the 3D defocus pattern of tracer particles.** **a** XZ Section through the cross-correlation reference library for the 3 μm polystyrene beads used in the experiments. Each row of pixels corresponds to one of 110 Z-levels, from -26.82µm to +27.32µm in 0.5µm steps. Dashed lines from top to bottom represent Z-positions at 16.39µm, 5.46µm, -4.97µm, and -15.89µm, with their corresponding reference library defocus patterns on the right. **b** Zoomed-in view of an XZ section through a preprocessed “median” stack. Rows represent frames in the reference video, bright vertical lines represent diffraction rings, and discontinuities in the vertical lines indicate change of the objective lens between Z-levels.

**Figure S3.**

## **Fig. S3 TrackMate errors increase with seed density.** a) Dropped spot rate (%). b) Linking error rate (average per particle).”

**Figure****S4.**

**Fig. S4** **Structure of the Deep learning model for particle Z-depth prediction**. Each block represents a convolution layer (*i.e.* Conv) with its kernel size and kernel number. The number below each block represents the number of times the layer is repeated.

**Figure S5.**

**Fig. S5** **Example synthetic particle image array for assessing XY localization accuracy (Z = 0 µm).** The array consists of particle images from the cross-correlation reference stack pasted at the nodes of a 15x19 array, equally spaced 128 pixels apart, onto a background of a particle-free image of the library generation circuit (*SI Appendix*, File S1). The XY σ was calculated by comparing positions predicted by TrackMate to the ground-truth paste locations.

**Figure****S6.**

**Fig. S6** **32x32 pixel reference stack images showing defocus patterns across the Z-range.** **a** Z = -26.82 µm, **b** -13.91 µm, **c** +13.91 µm, and **d** +27.32 µm. The defocus pattern at the negative extreme is roughly circular and centered within the frame, while the patterns become successively more elongated in the vertical (Y) direction at higher Z-levels. Aspect ratio (Y/X) of overlayed ellipse approximations to the central pattern are: 0.99, 1.04, 1.06, 1.10 for **a**-**d** respectively. The elongation can cause localization errors in XY that also result in off-center errors in the median-projected reference stack.

**Figure S7.**

**Fig. S7** **Histogram of the of Z-**$E_{prec}$ **for n = 265 pathlines from a flat Poiseuille flow.** Bin width 0.01 µm. Median Z-$E_{prec}$ values are 0.14 µm (Cross-Correlation) and 0.17 µm (Neural Network). Interquartile ranges are 0.06 µm for both histograms.

**Figure S8.**

**Fig. S8** **Reference image relabeling to account for device tilt.** **a** Example of an image used to extract reference particle images at Z = 0 µm. **b** Scatterplot of X values (as located by TrackMate) *vs.* Z values (as predicted by cross-correlation) for all particle images (n = 1,062) extracted from all 14 reference images at Z = 0 µm. The slope (-0.0028 µm/µm) reveals a tilt of the microfluidic device. Reference particle images at all positions are re-labeled to account for the slope using a re-binning process. A particle’s label is re-binned based on its horizontal X-position and the corresponding Z-position predicted by the linear fit. Given the objective Z-step size of ΔZ = 0.5 µm, all particles for which the fitted Z-position falls within +/- 0.25 µm from an original label are re-binned under that label. Thus, all particle images that fall within the vertical lines shown here are re-labeled at the Z-value of the dashed horizontal line contained within.

**Figure S9.**

**Fig. S9** **Side (XZ plane) views of pathlines extracted from the channel step dataset.** Z-predictions *via* **a** cross-correlation and **b** neural network. Sharp spikes in a single pathline reflect “gross” Z-prediction errors. One-off “spike” gross error can be easily handled with a median filter. Plateau errors, or sustained errors, as seen in **a** require filtering with a larger kernel size, which lowers spatial resolution. Low-amplitude “noise” variation is present along each pathline and we use the information to estimate the precision of both methods (see main text).

**Figure S10.**

**Fig. S10** **Kernel density estimations (KDE) of the probability density of** $\boldsymbol{\eta}$ **across the channel step velocity field**. Curves reflect results for fields lattice averaged using the element sizes listed in Table S4. Z-prediction via cross-correlation.

**Figure S11.**

**Fig. S11 Scatterplots of Δu/Δx *vs*. –(Δv/Δy + Δw/Δz) for the channel step dataset.** Element sizes used in the lattice averaging are in a) 1µm x 1µm x 1µm, b) 2µm x 2µm x 2µm, c) 4µm x 4µm x 4µm, and d) 4µm x 2µm x 1µm. Deviations from the 45° indicate continuity errors.

**Figure S12.**

**Fig. S12 Median Z-prediction times for cross-correlation and deep learning.** From a set of n=2,681 particle time-stacks with a median length of 53 frames. Error bars show the interquartile range.

**Figure S13.**

**Fig. S13** **Lattice averaged velocity vector fields calculated from the “Corrected” dataset.** Z-prediction *via* cross-correlation. **a**,**c** XY planar fields at **a** Z = 1.2 µm and **c** Z = 13.2 µm above the droplet’s horizontal mid-plane. Colormap represents velocity vector magnitude. Element size 4 µm x 2 µm x 4 µm (ΔX, ΔY, ΔZ). **b**,**d** Same as **a**,**c**, but with element size 2 µm x 1 µm x 4 µm. **e**,**f** Isometric views of the 3D velocity field, from the front/downstream end. **e** Colored for vector magnitude. **f** Colored for velocity out-of-plane (*w*) component).

## **Tables S1-S5**

**Table S1: Variation of dropped spot and linking error rates with the particle image density** $\boldsymbol{N}_{\boldsymbol{s}}$**.**

| Particle Image Density $N_{s}$ (pix/pix) | Number of spots | Dropped spots (%) | Linking Errors per Particle |
| --- | --- | --- | --- |
| 0.003 (0.27mg/ml) | 3461 | 0.8 | 0.08 |
| 0.007 (0.56 mg/ml) | 3487 | 1.2 | 0.19 |
| 0.009 (0.72 mg/ml) | 3702 | 2.3 | 0.84 |
| 0.028 (2.24 mg/ml) | 3553 | 2.6 | 1.99 |

**Table S2 Vector counts from droplets flowing in a straight channel.** Number of velocity vectors generated from the auto and corrected datasets and with our without filtering. The corrected dataset yields a higher number of velocity vectors. While the higher number is not significant enough to improve the spatial resolution, *i.e.* element size, it increases the recovery of data close to the interface.

| Dataset | Number of Vectors | Z-Prediction *via* | No Filter | 5th-order Median Filtered |
| --- | --- | --- | --- | --- |
|  |  |  | % Wmax-Filtered Vectors | % Wmax-Filtered Vectors |
| Auto | 21,777 | Cross-Correlation | 0.65 (99.35%) | 0.15 (99.85%) |
|  |  | Neural Network | 1.2 (98.8 %) | 0.34 (99.66%) |
| Corrected | 23,216  (+ 6.6%) | Cross-Correlation | 2.6 (104%) | 0.49 (106.1%) |
|  |  | Neural Network | 4.6 (102 %) | 1.2 (105.4%) |

**Table S3 Vector counts from droplets flowing in a curved channel.** Number of velocity vectors generated from the auto and corrected datasets and with our without filtering. The corrected dataset yields a higher number of velocity vectors. The results and conclusions are similar to the case of droplets flowing in a straight channel. The corrected dataset recovers mostly data next to the interface.

| Dataset | # of Vectors | Z-Prediction *via* | No Median Filter | 5th-order Median Filtered |
| --- | --- | --- | --- | --- |
|  |  |  | % Wmax-Filtered Vectors | % Wmax-Filtered Vectors |
| Auto | 21,566 | Cross-Correlation | 2.0  (98%) | 0.8  (99.2%) |
|  |  | Neural Network | 1.5  (98.5%) | 0.3  (99.7%) |
| Corrected | 24,226  (+ 12.3%) | Cross-Correlation | 1.5  (109.8%) | 0.5  (111.8%) |
|  |  | Neural Network | 4.4  (107.9%) | 1.4  (110.9%) |

**Table S4: Fluid properties of dispersed and continuous phases.** Dispersed phase is also used in single-phase experiments.

| Phase | Composition (% v/v) | Refractive  Index *n* | Density $\rho$ (g/cm^3) | Dyn. Viscosity $\mu$ (cP) | Interfacial Tension $\gamma$ (mN/m) |
| --- | --- | --- | --- | --- | --- |
| Bead suspension (Dispersed) | Water/Glycerol,  45/55 | 1.409 | 1.16 | 7.5 | 36 |
| Continuous | F6H8/Mineral Oil,  31.9/68.1 | 1.427 | 0.98 | 11.2 |  |

**Legends for Movies S1-S3**

## **Movie S1 (separate .avi file).** Droplet in a straight channel. The trajectory of the yellow circled bead in the lower left quadrant underlines the presence of a vortex close to the top interface (represented in blue in Fig. 5). The movie was taken at 500 fps and slowed down 50 fold.

**Movie S2 (separate .avi file).** Droplet in a curved channel. The trajectory of the yellow circled bead in the upper left quadrant underlines the presence of a vortex close to the top interface (represented in blue in Fig. 7). The movie was taken at 300 fps and slowed down 30 fold.

**Movie S3 (separate .avi file).** Droplet in a curved channel. The trajectory of the yellow circled bead in the right quadrant underlines the transition between the larger recirculation vortex and the smaller corner vortex (represented in yellow, pink and green respectively in Fig. 7). The movie was taken at 300 fps and slowed down 30 fold.

**Legends for Files S1-S12**

**Supplementary File S1 (separate .dwg file).** CAD designs used in this report. Data recorded at features as labeled. (A) Reference image capture circuit. Single layer, 50 µm target height. (B) Displacement structures circuit. 2-Layer, 11 µm (first layer) and 19 µm (second layer) target heights. (C) Droplet circuit. Single layer, 38 µm target height.

**Supplementary Files S2-S12 (separate .xml files).** The complete pathline-level datasets described in this report. Generated *via* TrackMate using the “Export tracks to XML file” function. Importable to MATLAB using the “importTrackMateTracks.m” MATLAB function included with TrackMate.

File S2: Displacement structures.

File S3: Straight droplets, Auto dataset. Z-prediction *via* cross-correlation.

File S4: Straight droplets, Corrected dataset. Z-prediction *via* cross-correlation.

File S5: Straight droplets, Auto dataset. Z-prediction *via* the deep learning model.

File S6: Straight droplets, Corrected dataset. Z-prediction *via* the deep learning model.

File S7: Curved droplets, Auto dataset. Z-prediction *via* cross-correlation.

File S8: Curved droplets, Corrected dataset. Z-prediction *via* cross-correlation.

File S9: Curved droplets, Auto dataset. Z-prediction *via* the deep learning model.

File S10: Curved droplets, Corrected dataset. Z-prediction *via* the deep learning model.

File S11: Channel step, Auto dataset. Z-prediction *via* cross-correlation.

File S12: Channel step, Auto dataset. Z-prediction *via* the deep learning model.

## **SI References**

1	Sauzade, M., Li, L., Bakowski, T., Strey, H. H. & Brouzes, E. Multi-layering of SU-8 exhibits distinct geometrical transitions from circular to planarized profiles. *Biomicrofluidics* **14**, 014116, doi:10.1063/1.5139031 (2020).

2	Kinoshita, H., Kaneda, S., Fujii, T. & Oshima, M. Three-dimensional measurement and visualization of internal flow of a moving droplet using confocal micro-PIV. *Lab Chip* **7**, 338-346, doi:10.1039/b617391h (2007).

3	Berry, J. D., Neeson, M. J., Dagastine, R. R., Chan, D. Y. & Tabor, R. F. Measurement of surface and interfacial tension using pendant drop tensiometry. *J Colloid Interface Sci* **454**, 226-237, doi:10.1016/j.jcis.2015.05.012 (2015).

4	Danjou, N. AMCap v. 9.3.29.4.

5	Mogne, A. D. a. A. Pendent_Drop: An ImageJ Plugin to Measure the Surface Tension from an Image of a Pendent Drop. *Journal of Open Research Software* **4**, doi:10.5334/jors.97 (2016).

6	Scanga, R. *et al.* Click Chemistry Approaches to Expand the Repertoire of PEG-based Fluorinated Surfactants for Droplet Microfluidics. *RSC advances* **8**, 12960-12974, doi:10.1039/c8ra01254g (2018).

7	Tinevez, J.-Y. *et al.* TrackMate: An open and extensible platform for single-particle tracking. *Methods* **115**, 80-90, doi:10.1016/j.ymeth.2016.09.016 (2017).

8	Arthur Miranda Neto, A. C. V., Isabelle Fantoni, Douglas & Eduardo Zampieri, J. V. F., Danilo Alves Lima. Image Processing Using Pearson’s Correlation

Coefficient: Applications on Autonomous Robotics. *13th International Conference on Mobile Robots and Competitions*

*(Robotica 2013)*, 14-19 (2013).

9	Krimmer, J. Quiver - magnitude-dependent color in 2D and 3D. *MATLAB Central File Exchange* (2021).

10	J. Zhang, B. T., J. Katz. Turbulent flow measurement in a square duct with hybrid holographic PIV. *Experiments in Fluids*, 373-381 (1997).
